# Comparative quality, readability, and factual reliability of four publicly accessible chatbot systems for heart failure patient education: a cross-sectional evaluation

**DOI:** 10.3389/fpubh.2026.1966612

**Published:** 2026-10-15

**Authors:** Nana Qin, Xinran Shao, Jing Wang, Jing Lei, Ran Wen, Wenjun Wu, Fangxu Liu, Hongwei Wang, Yan Zhu

**Affiliations:** 1 General Hospital of Northern Theater Command, Shenyang, Liaoning, China; 2 Changchun University of Chinese Medicine, Changchun, Jilin, China; 3 China Medical University, Shenyang, Liaoning, China; 4 Liaoning University of Traditional Chinese Medicine, Shenyang, Liaoning, China; 5 Shaanxi Provincial People’s Hospital, Xi’an, Shaanxi, China; 6 Tongchuan Traditional Chinese Medicine Hospital, Xi’an, Shaanxi, China

**Keywords:** chatbots, hallucination, heart failure, large language models, patient education, readability

## Abstract

**Background:**

Large language models (LLMs) are increasingly used for health information delivery, but comparative evidence on their quality, readability, and factual reliability in heart failure (HF) education remains limited.

**Objective:**

To compare four publicly accessible chatbot systems under prespecified access and retrieval configurations in answering common heart failure–related questions.

**Methods:**

We evaluated 108 standardized English HF questions, yielding 432 responses. Content quality was assessed using modified DISCERN (mDISCERN) and Ensuring Quality Information for Patients (EQIP), readability was assessed using established English-language readability indices, including the Simple Measure of Gobbledygook (SMOG), Flesch Reading Ease Score (FRES), Flesch–Kincaid Grade Level (FKGL), Automated Readability Index (ARI), Gunning Fog Index (GFOG), and Coleman-Liau Index (CLI), and factual reliability was evaluated by auditing all responses against the 2022 AHA/ACC/HFSA guideline and relevant clinical evidence. Continuous outcomes were analyzed using linear mixed-effects models. Hallucination outcomes were defined as clinically meaningful statements that were inconsistent with guidelines, unsupported by evidence, overgeneralized beyond the relevant population, or potentially misleading for patient education, and were analyzed using Wilson 95% confidence intervals (CIs), Cochran’s Q test, and generalized estimating equation (GEE) logistic regression.

**Results:**

DeepSeek generally showed the most favorable content-quality profile across the evaluated domains. In the primary readability analysis, Perplexity generated the most complex text, whereas sensitivity analysis using a second calculator showed partially different rankings. Hallucinations were identified in 6/108 (5.56%) DeepSeek responses, 8/108 (7.41%) ChatGPT responses, 10/108 (9.26%) Copilot responses, and 16/108 (14.81%) Perplexity responses. Paired binary analyses showed significant differences among chatbot systems (Cochran’s Q *p* = 0.002; GEE *p* = 0.006).

**Conclusion:**

Under the tested conditions and evaluated model versions, DeepSeek generally showed the most favorable content-quality profile and the lowest observed hallucination rate. However, hallucinations occurred across all systems, and pairwise factual-reliability differences were mainly driven by the higher hallucination rate of Perplexity. Guideline-aligned verification and clinician oversight remain necessary before patient-facing use.

## 1 Introduction

The rapid proliferation of the internet has fundamentally transformed patient education, with web-based resources increasingly becoming a major source of consumer health information (1). The quality and readability of such online resources have been extensively investigated over the past two decades (2). In parallel, the digital health landscape has been reshaped by artificial intelligence (AI), including LLM-driven systems that increasingly mediate health communication, clinical information delivery, and patient-facing health advice (3–6).

Heart failure (HF) is a chronic clinical syndrome caused by structural or functional cardiac abnormalities, presenting with symptoms such as dyspnea, fatigue, and fluid retention (7, 8). It remains a major global public health challenge due to its high prevalence, hospitalization burden, and mortality (9). Approximately 64 million individuals worldwide are affected (9). In China, nationally representative data have also shown a substantial HF burden among adults, particularly in older populations and those with cardiovascular comorbidities (10). Effective management requires long-term self-care, adherence to complex treatment regimens, and sustained patient education, which may improve self-care behaviors, quality of life, and clinical outcomes in patients with HF (11–13). However, evidence consistently shows that limited literacy and HF-related health literacy gaps are associated with poorer knowledge, self-care behaviors, quality of life, and clinical outcomes in patients with HF (14–16).

AI-enabled search engines and chatbots now offer a potential avenue to address these gaps. While prior studies have evaluated the quality and readability of AI-generated educational content, most have focused on single models, and few have examined their performance specifically in heart failure. Recent findings suggest that GPT-3.5 and GPT-4 can provide accurate and reliable answers to many heart failure-related questions (17). Yet, the comparative performance of different LLMs in delivering patient-centered, accessible, and reliable heart failure education remains poorly understood, and recent reviews have emphasized heterogeneity in reporting, human evaluation, and safety assessment across chatbot health advice studies (5, 18).

To address this gap, we conducted a systematic comparison of four widely used LLM-based chatbot systems, namely ChatGPT, DeepSeek, Perplexity, and Copilot, in answering common heart failure questions. Using a multidimensional evaluation framework encompassing content quality, readability, and factual reliability, this study aims to inform the potential integration of LLM-based chatbot systems into patient education and digital health strategies (6, 18).

## 2 Materials and methods

### 2.1 Question selection and classification

This was a cross-sectional, comparative methodological evaluation of publicly accessible LLM-based chatbot systems for patient-facing heart failure education. No human participants, patient records, or clinical interventions were involved. We used the complete set of 108 HF-related questions reported in a previous patient-education study (17). The complete question set was retained without adding, removing, or rewording individual questions. The questions were reviewed and categorized with reference to authoritative HF patient-education and guideline sources, including the American Heart Association Heart Failure Tools and Resources (19) and the 2023 focused update of the European Society of Cardiology HF guidelines (20). Because the complete predefined question set was included, no formal *a priori* sample-size calculation was performed. The questions were categorized into three clinically relevant domains: basic knowledge, management, and other HF-related issues.

The basic knowledge domain (*n* = 50) covered general definitions, symptoms, causes, pathophysiology, diagnostic tests, and HF classifications. The management domain (*n* = 41) addressed pharmacologic therapy, lifestyle modification, self-monitoring, rehabilitation, medication adherence, and routine self-care topics such as sodium and fluid restriction. The other domain (*n* = 17) included questions related to prognosis, invasive procedures, device-based therapies, psychosocial support, and family impact.

The categorization was based on clinical focus and patient-education relevance. To ensure clinical appropriateness, the final question classification was reviewed by two cardiologists and one heart failure specialist nurse. The complete list of the 108 standardized HF questions is provided in Supplementary Appendix A.

### 2.2 Chatbot selection and testing protocol

Four widely used LLM-based chatbot systems, namely ChatGPT, DeepSeek, Perplexity AI, and Microsoft Copilot, were evaluated using a standardized testing protocol. ChatGPT and DeepSeek were tested with web browsing disabled or unavailable, whereas Perplexity and Copilot were evaluated using their default online retrieval modes. All queries were conducted in Shenyang, Liaoning Province, China, between 1 and 5 August 2025 using the same workstation, physical network, IP connection, and operating system. Google Chrome v126.0 was used to access ChatGPT, DeepSeek, and Perplexity, whereas Microsoft Edge v127.0 was used for Copilot. The installed browser versions were retained throughout data collection, and automatic browser updates were disabled during the testing period.

Each of the 108 standardized heart failure-related questions was submitted in English using an identical prompt template: “Please answer the following question clearly and accurately for a patient audience: [question]. Keep your response concise, organized, and easy to understand.” No model-specific prompt optimization, follow-up questioning, or iterative refinement was performed. Each query was conducted in a single-turn, context-isolated session using a new session, fresh tab, or platform-specific context-reset procedure to minimize carry-over. The reporting of prompt structure, session control, access conditions, and retrieval status was aligned with recent recommendations for chatbot health advice studies (21).

Because web-based chatbot interfaces differ in the extent to which model versions and generation parameters are disclosed or configurable, all accessible settings were kept at default or comparable values whenever possible and documented for transparency. Detailed information on model identifiers, access tiers, browsing/retrieval status, session-handling procedures, operating environment, and available generation settings is provided in Supplementary Tables S1, S2. These reporting elements are intended to improve reproducibility in accordance with emerging guidance for chatbot health advice studies (21). No ethical approval was required because no human participants, personal data, or patient records were involved.

### 2.3 Factual accuracy and hallucination audit

All generated responses were cross-verified against the 2022 AHA/ACC/HFSA Heart Failure Guideline and relevant clinical evidence (22). Relevant clinical evidence included major contemporary heart failure guidelines and authoritative patient-education sources used during question-set refinement, including the AHA Heart Failure Tools and Resources and the 2023 focused update of the ESC heart failure guidelines (19, 20). When a statement was not directly addressed by the 2022 AHA/ACC/HFSA guideline, reviewers assessed whether it was supported by consistent contemporary guideline-based or peer-reviewed clinical evidence. Statements were classified as hallucinated only when they were clinically meaningful and guideline-inconsistent, contradicted established clinical evidence, were unsupported by applicable evidence, were overgeneralized beyond the relevant patient population, or could mislead patient-facing HF education. Because four chatbot systems each answered 108 standardized heart failure-related questions, all 432 generated responses were included in the accuracy and hallucination audit.

Hallucination was assessed at the response level. A response was classified as containing a hallucination if it included at least one clinically meaningful statement meeting the definition above. The hallucination rate was calculated as the proportion of responses containing at least one hallucinated statement among all responses generated by each system. This assessment focused on potential safety concerns arising from inaccurate or misleading medical information generated by chatbots and did not evaluate real-world clinical outcomes, patient harm, or changes in clinical decision-making. Because hallucination assessment and clinical safety represent different concepts, hallucination was evaluated as a separate safety-related outcome reflecting potential risks associated with misinformation rather than a direct measure of clinical safety outcomes. Expert clinical review was used for hallucination classification (18, 23, 24). Potential safety-related severity of hallucinated statements was categorized as low, moderate, or high. Low severity indicated an imprecise, unsupported, or overgeneralized statement unlikely to cause immediate harm but capable of reducing educational accuracy. Moderate severity indicated a statement that could plausibly influence medication use, supplement use, treatment expectations, care-seeking behavior, or self-management decisions if followed without clinician review. High severity indicated a statement that could reasonably encourage unsafe action, delay emergency care, or contradict core guideline-directed treatment.

Two independent reviewers with cardiovascular surgical training performed the hallucination audit (Baoyin Li and Fangxu Liu). Disagreements were resolved through discussion, and unresolved cases were adjudicated by a senior cardiologist (Yan Zhu). Inter-rater agreement for hallucination classification was assessed using Cohen’s kappa (23). For each model, hallucination rates were reported as counts, percentages, and Wilson 95% confidence intervals. Because each HF question was answered by all four chatbot systems, between-system differences in hallucination outcomes were assessed using paired binary methods. A generalized estimating equation logistic regression model with question ID as the clustering variable was used as a sensitivity analysis to account for question-level correlation (25). Cochran’s Q test was used for the overall paired comparison across the four systems, and exact pairwise McNemar tests with Bonferroni adjustment were used for pairwise comparisons (26). Response-level hallucination rates by model and question domain are summarized in Supplementary Table S5, the full response-level hallucination audit is provided in Supplementary Table S6, and paired binary analyses are reported in Supplementary Table S7.

### 2.4 Content quality assessment

Responses generated by the four chatbots were independently evaluated by three raters: two authors with cardiovascular surgery backgrounds, Baoyin Li and Fangxu Liu, and one heart failure specialist nurse, Hongwei Wang. All raters assessed the responses using two predefined patient-information quality instruments: the modified DISCERN (mDISCERN) and the Ensuring Quality Information for Patients (EQIP) tools.

To minimize assessment bias, all chatbot responses were anonymized and coded as Models A-D before scoring. The raters were blinded to chatbot identity, and all responses were presented in randomized order. Each rater scored the responses independently according to the predefined scoring criteria. After independent scoring, disagreements or uncertain ratings were discussed to reach consensus. Any unresolved discrepancies were adjudicated by a senior cardiologist, Yan Zhu. Although complete masking of model identity could not be guaranteed because of model-specific linguistic styles, anonymization and randomized presentation were used to reduce systematic bias.

The mDISCERN tool used in this study was adopted from a previously published modified DISCERN instrument based on the original DISCERN questionnaire, which was developed to assess the reliability and quality of written consumer health information regarding treatment choices (27–29). Because the original DISCERN instrument was designed for conventional written materials and includes items related to publication sources and references, some items were not directly applicable to AI-generated chatbot responses. Following previous studies evaluating AI-generated health information, source-dependent items that could not be reliably assessed in chatbot-generated responses were excluded (30).

The resulting 12-item modified DISCERN instrument retained the core domains relevant to LLM-generated patient information, including reliability, clarity of treatment options, balance, and overall information quality. Each mDISCERN item was rated on a 5-point scale ranging from 1 to 5, and the final mDISCERN score was calculated as the mean of the 12 retained item scores, with higher scores indicating better information quality. The complete scoring template and retained items are provided in Supplementary Appendix B for transparency.

The EQIP tool was used to evaluate the clarity, structure, completeness, and practical usefulness of patient-directed information (31). EQIP consists of 20 items covering key features of written patient information, including organization, understandability, balance, and actionability. Each item was scored as “Yes,” “Partially,” or “No.” In this study, these responses were assigned values of 1, 0.5, and 0, respectively. The final EQIP percentage score was calculated as follows: Score = [(X × 1) + (Y × 0.5) + (Z × 0)] / (20 – Q) × 100, where X, Y, and Z represent the numbers of “Yes,” “Partially,” and “No” responses, respectively, and Q represents the number of “not applicable” items. Scores were categorized as poor quality (0–50%), good quality with minor deficiencies (51–75%), or excellent quality (76–100%) (31, 32). The detailed scoring templates for mDISCERN and EQIP are provided in Supplementary Appendix B for transparency and reproducibility.

It should be noted that mDISCERN and EQIP primarily assess the quality, structure, completeness, and patient-oriented presentation of health information rather than factual accuracy or clinical safety (27, 31, 33). Therefore, factual accuracy and hallucination assessment were conducted separately against the 2022 AHA/ACC/HFSA Guideline for the Management of Heart Failure, as described in Section 2.3. Inter-rater reliability for mDISCERN and EQIP scoring was assessed using a two-way random-effects intraclass correlation coefficient for absolute agreement based on the mean ratings of multiple raters [ICC(2,k)] (34).

### 2.5 Readability analysis

Readability of the chatbot-generated responses was assessed using established English-language readability formulas. Before analysis, non-response elements that could affect automated readability calculations, including copied prompts, interface labels, hyperlinks, reference markers, and duplicated source lists, were removed where applicable. The remaining patient-facing response text was then analyzed using two online readability calculators: ReadabilityFormulas.com (Calculator 1: http://readabilityformulas.com/) and Online-Utility.org (Calculator 2: https://www.online-utility.org/english/readability_test_and_improve.jsp).

The following established English-language readability indices were calculated: Coleman-Liau Index (CLI), Automated Readability Index (ARI), Simple Measure of Gobbledygook (SMOG), Gunning Fog Index (GFOG), Flesch Reading Ease Score (FRES), and Flesch–Kincaid Grade Level (FKGL). Calculator 1 was used as the primary analysis tool because it provides several widely used readability indices for English-language patient education materials, including FRES, FKGL, GFOG, ARI, and SMOG. Calculator 2 was used as a secondary sensitivity tool to examine whether the relative readability patterns among models were consistent under an alternative algorithmic implementation.

SMOG was prespecified as the primary readability outcome because it is commonly used to estimate the reading grade level of patient-oriented health information. FKGL and ARI were treated as secondary grade-level indices, whereas FRES was used as an ease-of-reading measure, with higher FRES scores indicating easier readability. Results from Calculator 1 were used for the main readability comparison, and results from Calculator 2 were used to assess robustness across different tokenization, sentence segmentation, syllable counting, and rounding rules.

### 2.6 Language and applicability of readability metrics

All prompts and chatbot outputs were generated and analyzed in English. Each question was entered verbatim in English, and all model responses included in the readability analysis were obtained as English-language text. The readability indices used in this study, including the ARI, FRES, GFOG, FKGL, SMOG, and CLI, were selected because they are widely used for evaluating English-language health information and patient education materials (35–38).

These formulas estimate readability based on English linguistic features, such as sentence length, word length, syllable count, and character count. Therefore, their results should be interpreted as indicators of English-language textual complexity and may not be directly generalizable to non-English or translated health information. To maintain methodological consistency, any non-answer elements or non-English interface artifacts were removed before readability calculation when applicable. No mixed-language responses were included in the final readability analysis.

### 2.7 Statistical analysis

Statistical analyses were performed using IBM SPSS Statistics version 24.0 (IBM Corp., Armonk, NY, USA) and R version 4.3.2 (R Foundation for Statistical Computing, Vienna, Austria). SPSS was used for descriptive statistics, inter-rater reliability analyses, and assessment of internal consistency. Data import and processing in R were performed using the readxl and dplyr packages. Linear mixed-effects models were fitted using the lme4 and lmerTest packages, estimated marginal means and Tukey-adjusted pairwise comparisons were obtained using the emmeans package, generalized estimating equation analyses were performed using the geepack package, Cochran’s Q tests were conducted using the DescTools package, and Wilson 95% confidence intervals were calculated using the binom package. Exact pairwise McNemar *p* values were obtained using exact binomial tests based on discordant response pairs. Figures were generated using GraphPad Prism version 8.4.0 (GraphPad Software, San Diego, CA, USA).

Continuous outcomes, including modified DISCERN (mDISCERN) scores, Ensuring Quality Information for Patients (EQIP) percentage scores, and readability indices, were summarized as mean ± standard deviation (SD). Because each of the 108 standardized questions was answered by all four chatbot systems, observations were treated as clustered at the question level. Continuous outcomes were therefore analyzed using linear mixed-effects models, with chatbot system specified as a fixed effect and question ID included as a random intercept (39). Models were fitted using maximum likelihood estimation. Separate analyses were conducted for the basic knowledge, management, and other HF-related question domains. When the overall chatbot-system effect was statistically significant, estimated marginal means were calculated, and pairwise comparisons between chatbot systems were performed using Tukey adjustment for multiple comparisons. EQIP scores were analyzed on a 0–100 percentage scale.

Hallucination was analyzed as a binary response-level outcome, as defined in Section 2.3. For each chatbot system, hallucination outcomes were summarized as counts, percentages, and Wilson 95% confidence intervals. Because the same set of 108 questions was evaluated across all four chatbot systems, analyses accounted for the paired and clustered structure of the data. A generalized estimating equation logistic regression model with an exchangeable working correlation structure and question ID as the clustering variable was performed as a sensitivity analysis to account for within-question correlation (25). DeepSeek was specified as the reference category, and the results were reported as odds ratios with 95% confidence intervals. Cochran’s Q test was used to assess the overall paired difference in hallucination outcomes across the four chatbot systems. Exact pairwise McNemar tests based on discordant response pairs were subsequently performed for *post-hoc* comparisons, with Bonferroni adjustment applied to account for the six pairwise comparisons (26).

Inter-rater reliability was assessed using the ratings obtained independently before consensus discussion or adjudication. Agreement between the two reviewers for binary hallucination classification was evaluated using Cohen’s κ (40). Agreement among the three raters for the mDISCERN and EQIP instruments was assessed using a two-way random-effects intraclass correlation coefficient for absolute agreement based on the mean ratings of multiple raters [ICC(2,k)] (34). ICC values below 0.50 were interpreted as indicating poor reliability, values from 0.50 to 0.75 as moderate reliability, values from 0.75 to 0.90 as good reliability, and values above 0.90 as excellent reliability. Internal consistency of the 12-item modified mDISCERN instrument was assessed using Cronbach’s α.

All statistical tests were two-sided, and *p* < 0.05 was considered statistically significant. *p* values were reported to three decimal places, with values below 0.001 reported as *p* < 0.001. All other statistical estimates, including means, SDs, test statistics, odds ratios, reliability coefficients, and confidence-interval limits, were reported to two decimal places.

## 3 Results

### 3.1 Content quality across models

Content-quality scores differed significantly among the four chatbot systems across all three question domains. In the mixed-effects models, the overall system effect was significant for both mDISCERN and EQIP in the basic knowledge, management, and other HF-related domains (all *p* < 0.001; Table 1; Figures 1A,B). DeepSeek generally achieved the highest mDISCERN scores, whereas Perplexity showed the lowest mDISCERN and EQIP scores across most domains.

**Table 1:** Comparative evaluation of content quality among the four LLM-based chatbot systems.

Domain	Metric	ChatGPT Mean ± SD	DeepSeek Mean ± SD	Perplexity Mean ± SD	Copilot Mean ± SD	*p* value
Basic knowledge (*n* = 50)	mDISCERN	3.26 ± 0.77ᵃᵇᶜ	3.73 ± 0.75ᵈᵉ	2.82 ± 0.75	3.01 ± 0.61	<0.001
Basic knowledge (*n* = 50)	EQIP (%)	53.69 ± 13.67ᵇ	57.85 ± 15.32ᵈ	45.20 ± 14.09ᶠ	53.72 ± 13.92	<0.001
Management (*n* = 41)	mDISCERN	3.88 ± 0.54ᵃ	4.36 ± 0.49ᵈ	3.78 ± 0.63ᶠ	4.12 ± 0.54	<0.001
Management (*n* = 41)	EQIP (%)	58.65 ± 11.81ᵃᶜ	65.41 ± 12.69ᵈ	55.01 ± 8.16ᶠ	64.43 ± 9.05	<0.001
Other HF-related issues (*n* = 17)	mDISCERN	3.87 ± 0.59ᵃᵇ	4.43 ± 0.27ᵈ	3.44 ± 0.50ᶠ	4.16 ± 0.27	<0.001
Other HF-related issues (*n* = 17)	EQIP (%)	53.59 ± 12.47ᵃᶜ	62.38 ± 11.19ᵈ	48.97 ± 8.25ᶠ	63.09 ± 7.05	<0.001

SD, standard deviation; mDISCERN, modified DISCERN; EQIP, Ensuring Quality Information for Patients. Except for *p* values, statistical estimates are reported to two decimal places. *p* values are reported to three decimal places, with values below 0.001 reported as *p* < 0.001. Overall *p* values are from linear mixed-effects models with chatbot system as a fixed effect and question ID as a random intercept. Pairwise comparisons were performed using Tukey adjustment. Superscript letters indicate statistically significant pairwise differences: a, ChatGPT vs. DeepSeek; b, ChatGPT vs. Perplexity; c, ChatGPT vs. Copilot; d, DeepSeek vs. Perplexity; e, DeepSeek vs. Copilot; f, Perplexity vs. Copilot.

**Figure 1:**
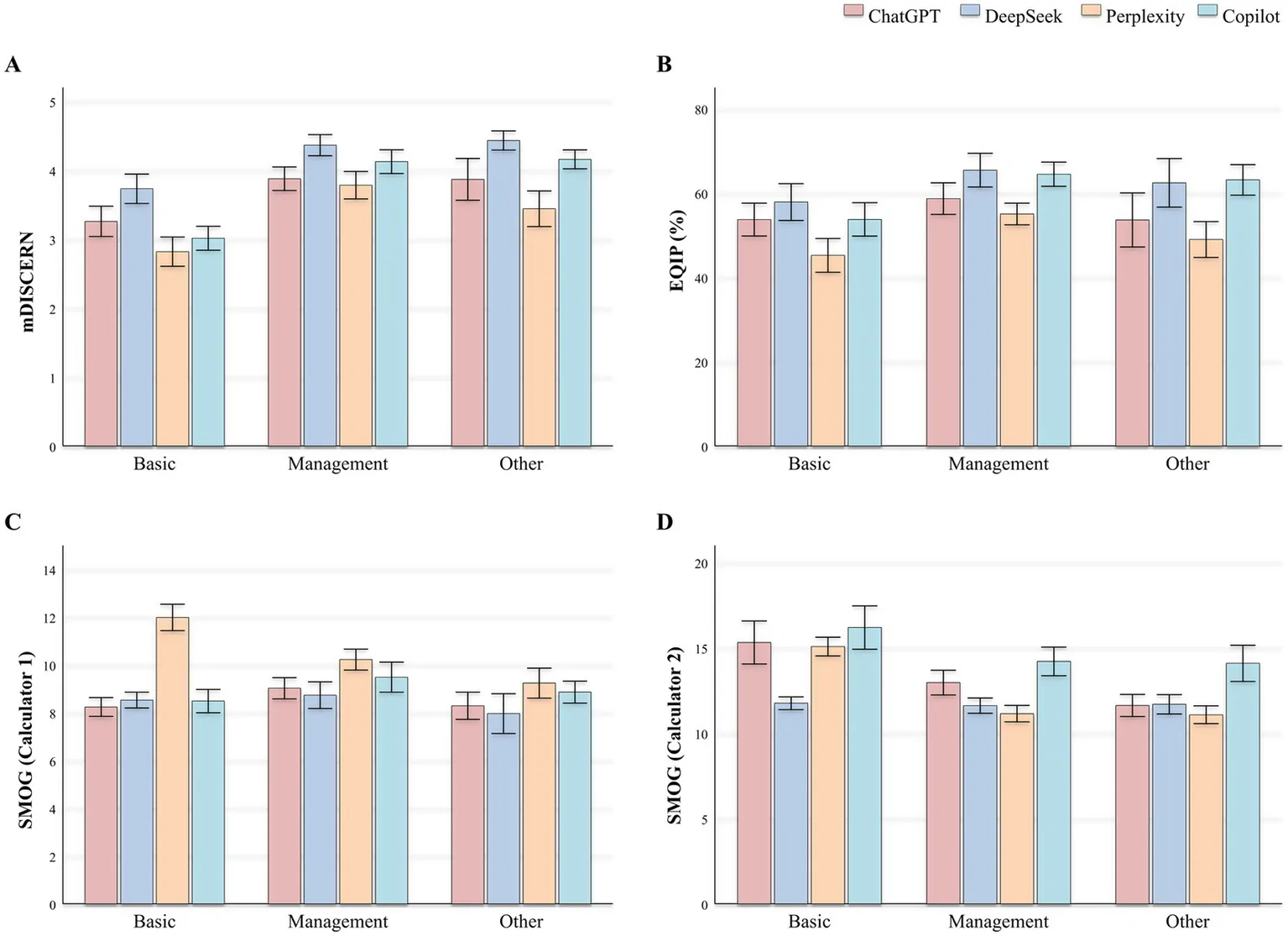
Comparisons of selected evaluation metrics across the four LLM-based chatbot systems, stratified by question category. Panels **A** and **B** show content-quality outcomes assessed using mDISCERN and EQIP, respectively. Panel **C** shows the primary SMOG readability results calculated using Calculator 1, and Panel **D** shows the sensitivity SMOG readability results calculated using Calculator 2. Bars represent mean values, and error bars represent 95% confidence intervals. Abbreviations: EQIP, Ensuring Quality Information for Patients; LLM, large language model; mDISCERN, modified DISCERN; SMOG, Simple Measure of Gobbledygook.

Tukey-adjusted pairwise comparisons indicated that the most consistent differences were observed between DeepSeek and Perplexity, with DeepSeek scoring significantly higher for both mDISCERN and EQIP across all domains. DeepSeek also showed higher mDISCERN scores than ChatGPT in all three domains, and Perplexity showed lower EQIP scores than DeepSeek and Copilot in all domains. In the basic knowledge domain, Tukey adjustment also identified a small but statistically significant mDISCERN difference between ChatGPT and Copilot. According to the predefined EQIP thresholds, the domain-level mean EQIP scores for ChatGPT, DeepSeek, and Copilot fell within the “good quality with minor deficiencies” range (51–75%) across all three domains. For Perplexity, the EQIP score in the management domain also fell within this range, whereas its scores in the basic knowledge domain and other HF-related domain fell within the “poor quality” range (0–50%). No domain-level mean EQIP score reached the “excellent quality” category (76–100%).

### 3.2 Primary readability analysis using calculator 1

Primary SMOG readability scores calculated using Calculator 1 differed significantly among the four chatbot systems in all three question domains (Table 2; Figure 1C). The overall mixed-effects model was significant for basic knowledge questions (*p* < 0.001), management questions (*p* < 0.001), and other HF-related questions (*p* = 0.004). Perplexity had the highest SMOG scores in all three domains, indicating greater linguistic complexity. Tukey-adjusted pairwise comparisons showed that Perplexity had significantly higher SMOG scores than ChatGPT, DeepSeek, and Copilot in the basic knowledge domain, and higher SMOG scores than ChatGPT and DeepSeek in the management domain. In the other HF-related domain, the overall model effect remained significant and the DeepSeek–Perplexity comparison was significant after Tukey adjustment.

**Table 2:** Primary SMOG readability scores calculated using Calculator 1 across the four LLM-based chatbot systems.

Domain	Metric	ChatGPT Mean ± SD	DeepSeek Mean ± SD	Perplexity Mean ± SD	Copilot Mean ± SD	*p* value
Basic knowledge (*n* = 50)	SMOG	8.24 ± 1.38ᵇ	8.53 ± 1.16ᵈ	11.98 ± 1.94ᶠ	8.48 ± 1.73	<0.001
Management (*n* = 41)	SMOG	9.02 ± 1.41ᵇ	8.73 ± 1.77ᵈ	10.22 ± 1.39	9.48 ± 1.99	<0.001
Other HF-related issues (*n* = 17)	SMOG	8.29 ± 1.11	7.96 ± 1.62ᵈ	9.23 ± 1.22	8.89 ± 0.89	0.004

SD, standard deviation; SMOG, Simple Measure of Gobbledygook. Except for p values, statistical estimates are reported to two decimal places. *p* values are reported to three decimal places, with values below 0.001 reported as *p* < 0.001. Overall *p* values are from linear mixed-effects models with chatbot system as a fixed effect and question ID as a random intercept. Pairwise comparisons were performed using Tukey adjustment. Superscript letters indicate statistically significant pairwise differences as defined in Table 1. Calculator 1: ReadabilityFormulas.com, http://readabilityformulas.com/.

Overall, Calculator 1 results indicated that Perplexity tended to generate the most linguistically complex responses, whereas the other systems produced comparatively more accessible text in this primary readability analysis. Secondary Calculator 1 readability indices, including ARI, FRES, GFOG, and FKGL, are provided in Supplementary Table S3 and Supplementary Figure S1.

### 3.3 Sensitivity readability analysis using calculator 2

Calculator 2 was used as a sensitivity analysis to evaluate whether readability differences persisted under an alternative algorithmic implementation. SMOG scores calculated using Calculator 2 differed significantly among the four chatbot systems across all three domains (all *p* < 0.001; Table 3; Figure 1D). Copilot had the highest SMOG scores in the basic knowledge, management, and other HF-related domains, whereas DeepSeek generally showed lower grade-level readability scores, particularly in the basic knowledge and management domains.

**Table 3:** Sensitivity SMOG readability scores calculated using Calculator 2 across the four LLM-based chatbot systems.

Domain	Metric	ChatGPT Mean ± SD	DeepSeek Mean ± SD	Perplexity Mean ± SD	Copilot Mean ± SD	*p* value
Basic knowledge (*n* = 50)	SMOG	15.31 ± 4.42ᵃ	11.75 ± 1.31ᵈᵉ	15.07 ± 1.92	16.18 ± 4.46	<0.001
Management (*n* = 41)	SMOG	12.96 ± 2.29ᵃᵇᶜ	11.61 ± 1.41ᵉ	11.14 ± 1.52ᶠ	14.20 ± 2.66	<0.001
Other HF-related issues (*n* = 17)	SMOG	11.62 ± 1.25ᶜ	11.69 ± 1.10ᵉ	11.07 ± 1.01ᶠ	14.09 ± 2.06	<0.001

SD, standard deviation; SMOG, Simple Measure of Gobbledygook. Except for p values, statistical estimates are reported to two decimal places. *p* values are reported to three decimal places, with values below 0.001 reported as *p* < 0.001. Overall *p* values are from linear mixed-effects models with chatbot system as a fixed effect and question ID as a random intercept. Pairwise comparisons were performed using Tukey adjustment. Superscript letters indicate statistically significant pairwise differences as defined in Table 1. Calculator 2: Online-Utility.org, https://www.online-utility.org/english/readability_test_and_improve.jsp.

These findings confirmed that readability differed across chatbot systems, although the rank ordering was not identical to that observed with Calculator 1. Therefore, Calculator 2 results should be interpreted as a sensitivity analysis rather than a direct replication of the primary readability analysis. Detailed Calculator 2 indices, including CLI, FKGL, and ARI, are provided in Supplementary Table S4 and Supplementary Figure S1.

### 3.4 Integrated performance summary

Across the main analyses, DeepSeek showed the most favorable content-quality profile, with higher mDISCERN and EQIP scores across the three question domains. The most consistent pairwise differences were observed between DeepSeek and Perplexity, with DeepSeek generally achieving higher content-quality scores. Readability findings were more dependent on the calculator and index used: Calculator 1 indicated greater linguistic complexity for Perplexity, particularly in the basic knowledge and management domains, whereas Calculator 2 suggested higher complexity for Copilot on SMOG-based sensitivity analysis. Overall, no chatbot system showed uniformly superior performance across all evaluation dimensions. DeepSeek showed the most favorable content-quality profile and the lowest observed hallucination rate under the tested conditions, but readability rankings varied according to the calculator and index used (Tables 1–3; Figures 1, 2).

**Figure 2:**
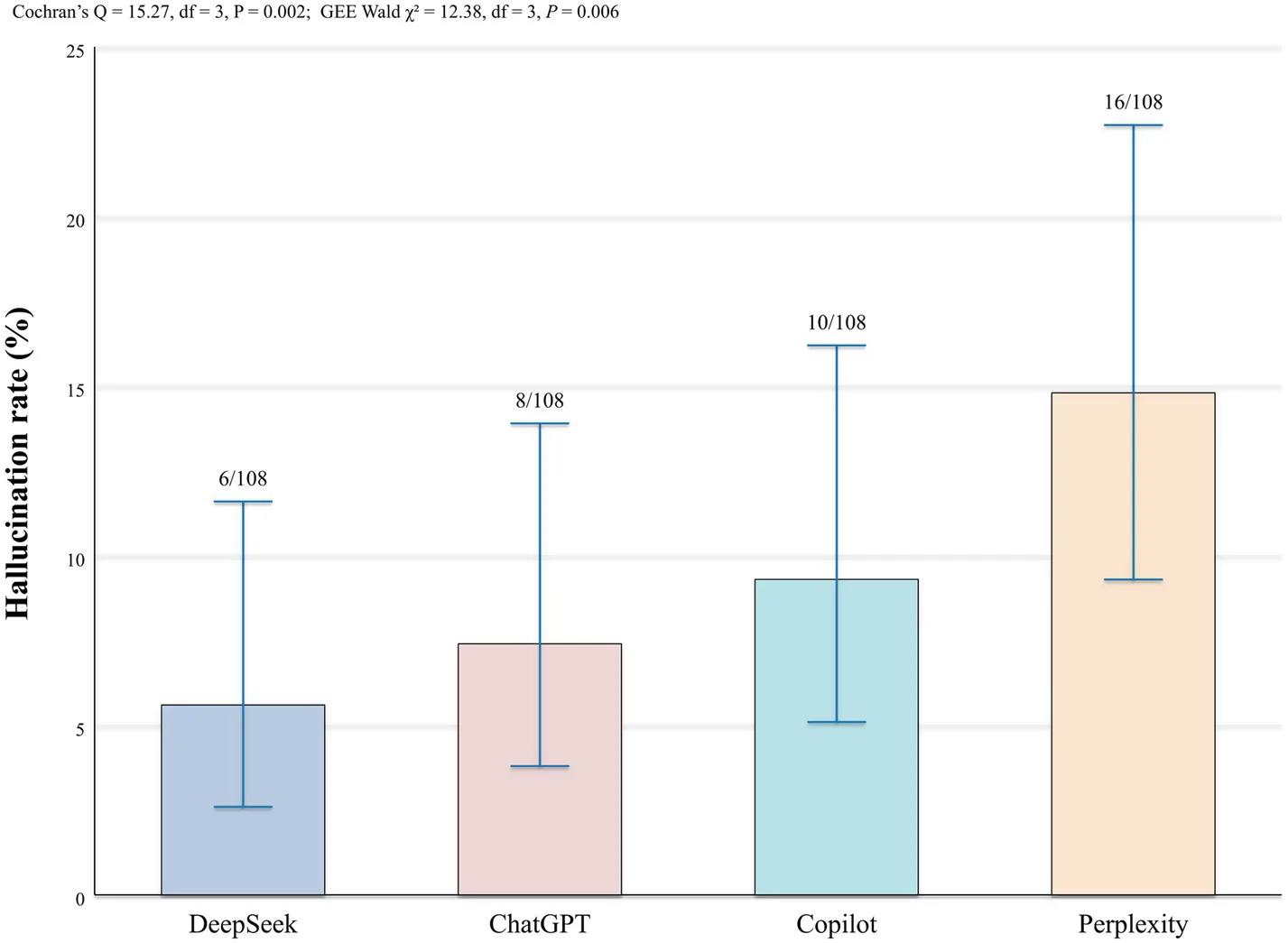
Response-level hallucination rates across the four LLM-based chatbot systems. Hallucination rates were calculated from a full audit of all 432 generated responses against the 2022 AHA/ACC/HFSA Heart Failure Guideline and relevant clinical evidence. A response was classified as hallucinated if it contained at least one clinically meaningful statement that was guideline-inconsistent, unsupported by applicable evidence, overgeneralized beyond the relevant patient population, or potentially misleading for patient-facing heart failure education. Chatbot systems are ordered by increasing hallucination rate. Error bars represent Wilson 95% confidence intervals. DeepSeek showed the lowest observed hallucination rate (6/108, 5.56%; 95% CI, 2.57–11.59%), followed by ChatGPT (8/108, 7.41%; 95% CI, 3.80–13.94%), Copilot (10/108, 9.26%; 95% CI, 5.11–16.21%), and Perplexity (16/108, 14.81%; 95% CI, 9.33–22.72%). Paired binary analysis showed a significant overall difference among chatbot systems by Cochran’s Q test (Q = 15.27, df = 3, *p* = 0.002), and generalized estimating equation logistic regression showed a similar overall system effect (Wald χ^2^ = 12.38, df = 3, *p* = 0.006). Abbreviations: AHA/ACC/HFSA, American Heart Association/American College of Cardiology/Heart Failure Society of America; CI, confidence interval; GEE, generalized estimating equation; LLM, large language model.

### 3.5 Reliability assessment and full factual accuracy audit

Inter-rater reliability for the human-rated content-quality instruments was high. The ICC values were 0.87 for mDISCERN and 0.83 for EQIP, indicating good agreement among the three raters. The modified mDISCERN instrument also demonstrated good internal consistency, with a Cronbach’s α of 0.87. Agreement between the two reviewers for response-level hallucination classification was also high, with a Cohen’s κ of 0.84.

A full response-level accuracy audit was conducted for all 432 generated responses against the 2022 AHA/ACC/HFSA Heart Failure Guideline and relevant clinical evidence. Overall, hallucinations were identified in 40 responses. DeepSeek showed the lowest hallucination rate (6/108, 5.56%; 95% CI, 2.57–11.59%), followed by ChatGPT (8/108, 7.41%; 95% CI, 3.80–13.94%), Copilot (10/108, 9.26%; 95% CI, 5.11–16.21%), and Perplexity (16/108, 14.81%; 95% CI, 9.33–22.72%; Figure 2).

Paired binary analysis showed significant differences in hallucination rates among the four chatbot systems. Cochran’s Q test demonstrated a significant overall difference (Q = 15.27, df = 3, *p* = 0.002), and the GEE logistic regression model accounting for question-level clustering showed a consistent overall model effect (Wald χ^2^ = 12.38, df = 3, *p* = 0.006). Using DeepSeek as the reference group, the odds ratios for producing a hallucinated response were 1.36 for ChatGPT (95% CI, 0.75–2.48; *p* = 0.317), 1.74 for Copilot (95% CI, 0.90–3.36; *p* = 0.103), and 2.96 for Perplexity (95% CI, 1.51–5.79; *p* = 0.002). Exact pairwise McNemar tests showed that the differences between ChatGPT and Perplexity (Bonferroni-adjusted *p* = 0.047) and between DeepSeek and Perplexity (Bonferroni-adjusted *p* = 0.012) remained significant after multiplicity correction. The identified hallucinations mainly involved guideline-inconsistent statements, unsupported clinical claims, overgeneralized treatment recommendations, and potentially misleading treatment-related information. No high-severity hallucinations were identified; all hallucinated responses were classified as low or moderate severity. Complete response-level classifications, severity ratings, audit rationales, and reference standards are provided in Supplementary Table S6, and detailed paired binary analyses are reported in Supplementary Table S7.

## 4 Discussion

In this study, we systematically compared four widely used LLM-based chatbot systems, namely ChatGPT, DeepSeek, Perplexity, and Copilot, in responding to standardized HF-related questions. By combining human-rated patient-information quality instruments, automated readability assessment, and a full response-level hallucination audit, we evaluated complementary dimensions of chatbot-generated HF education content, including information structure, accessibility, and factual reliability. The findings showed that chatbot performance varied substantially across evaluation dimensions. DeepSeek generally demonstrated the most favorable content-quality profile and the lowest observed hallucination rate, whereas Perplexity tended to generate more linguistically complex responses in the primary readability analysis and showed the highest hallucination rate. However, readability rankings differed between calculators, and differences in hallucination rates should not be interpreted as demonstrating superiority in clinical safety outcomes for any system. These results highlight that LLM performance in patient education should be assessed multidimensionally rather than inferred from fluency, response length, or apparent informativeness alone (5, 6, 17).

Although this study uses the term “content quality,” mDISCERN and EQIP primarily evaluate the presentation, organization, balance, and completeness of patient information rather than factual correctness or direct clinical safety outcomes (27, 29). These instruments assess whether health information is clear, structured, actionable, and supportive of patient decision-making, but they do not independently verify whether each clinical statement is guideline-concordant. The adapted 12-item mDISCERN used in this study preserved the core conceptual domains of the original DISCERN instrument, including reliability, clarity of treatment options, and overall quality of information (27, 29). Previous studies have shown that shortened DISCERN-based instruments can help identify evidence-based online health information, although the adapted 12-item version used in the present study should be interpreted as a study-specific adaptation rather than a fully independent validation (29, 41).

Factual accuracy was therefore evaluated separately through a full response-level hallucination audit of all 432 generated responses against the 2022 AHA/ACC/HFSA Heart Failure Guideline and relevant clinical evidence. Hallucinated responses were identified in all four chatbot systems, indicating that structured and readable responses may still contain clinically unsupported or potentially misleading statements. In paired analyses that accounted for the shared question set across systems, hallucination rates differed significantly among the four chatbots, with Perplexity showing the highest rate and DeepSeek the lowest observed rate. Pairwise differences were mainly driven by the higher hallucination rate of Perplexity, and no chatbot system was free from hallucinated responses. These findings should be interpreted in the context of the relatively small number of hallucination events and the clinical judgment required for response-level classification. Nevertheless, they reinforce the need for guideline-aligned verification, explicit safeguards against misinformation, and clinician oversight before LLM-generated HF information is used in patient-facing settings, particularly for medication use, symptom escalation, device therapy, and procedural decision-making (23, 24).

DeepSeek achieved higher mDISCERN and EQIP scores than the other chatbot systems in most domains, particularly for management-related questions. This may reflect its tendency to provide more structured, patient-oriented responses that included explanations of treatment options, potential benefits and risks, self-care advice, and indications for seeking medical attention. These elements correspond closely to the domains emphasized by mDISCERN and EQIP and may therefore have contributed to its higher evaluator-rated content-quality scores. Previous reviews have suggested that domain-specific adaptation, curated medical knowledge integration, and retrieval-augmented approaches may improve the completeness, reliability, and evidence-based reasoning of LLM-generated clinical content (6, 42). However, the present study did not examine the training data, fine-tuning strategy, retrieval architecture, or internal reasoning mechanisms of the evaluated systems. Therefore, the observed differences should be interpreted as comparative performance under the tested conditions rather than evidence of specific model-design advantages.

Response structure may also have influenced evaluator-rated performance. Under the standardized prompt used in this study, DeepSeek more frequently presented information in a stepwise format, with contextual explanations, practical recommendations, and safety alerts. Such organization may improve perceived completeness and usability, especially for HF management questions that require action-oriented guidance. Prior work has emphasized that model configuration, prompting strategy, and output design can influence the clarity, organization, and usability of LLM-generated health information (6, 21). Because all chatbot systems received the same standardized prompt and no model-specific prompt optimization was performed, the observed differences likely reflect system-specific response behavior under the tested conditions.

The readability findings further demonstrate that patient-facing LLM outputs should be evaluated beyond content completeness alone. In the primary analysis using Calculator 1, Perplexity generated responses with higher grade-level readability scores, suggesting greater linguistic complexity than the other chatbot systems. However, the alternative Calculator 2 analysis produced a different rank ordering, indicating that readability estimates are partly dependent on the formula, calculator, and underlying text-processing procedures used. These findings are clinically relevant because HF education commonly targets older adults and individuals with limited health literacy, for whom complex language may impair comprehension of medication use, dietary and fluid recommendations, daily weight monitoring, symptom escalation, and thresholds for seeking urgent care (14–16). Previous studies have similarly shown that many hospital-issued and web-based patient education materials exceed recommended readability levels, while guidance generally supports targeting approximately a sixth- to eighth-grade reading level to improve accessibility and patient engagement (38, 43–45). Although technically detailed responses may benefit readers with greater medical literacy, patient-facing LLM outputs should balance clinical precision with linguistic accessibility and provide concise, actionable, and safety-oriented guidance.

Readability scores should be interpreted as comparative indicators of textual complexity rather than direct measures of patient comprehension. ReadabilityFormulas.com and Online-Utility.org use different procedures for tokenization, sentence segmentation, syllable counting, and rounding, which may explain differences in absolute values and rank ordering across calculators. Calculator 1 was therefore used for the primary readability analysis, whereas Calculator 2 was used as a sensitivity analysis. Although the two calculators did not produce identical rankings across all indices and domains, both supported the broader conclusion that readability varied meaningfully across chatbot systems.

ChatGPT and Copilot showed intermediate overall performance across the evaluated domains. ChatGPT produced generally coherent and clinically relevant responses but did not consistently achieve the highest mDISCERN or EQIP scores, whereas Copilot showed mixed performance across readability and content-quality outcomes. These findings are consistent with prior evidence that LLM performance can vary substantially according to task type, prompt design, and evaluation context, and that performance on structured medical examinations may not directly translate into optimal patient-facing communication (46, 47). Readable presentation may improve accessibility, but it cannot substitute for structured, comprehensive, and guideline-concordant information.

The multidimensional evaluation framework used in this study has practical implications for future assessment of healthcare LLMs. Expert-rated instruments such as mDISCERN and EQIP assess organization, balance, actionability, treatment-related information, and support for shared decision-making, while readability metrics estimate textual complexity and hallucination auditing evaluates factual reliability and potential safety risks related to misinformation. These dimensions are complementary rather than interchangeable. Therefore, healthcare LLMs should not be evaluated using a single metric, such as fluency, perceived completeness, or readability alone. This approach is consistent with recent recommendations that medical LLMs be assessed using multidimensional frameworks incorporating human evaluation and safety-related assessments (6, 18). However, neither expert scoring nor readability formulas directly measure patient comprehension, clinical decision-making, behavioral change, or clinical outcomes (48, 49). Future studies should incorporate real-world patient testing, scenario-based comprehension tasks, multi-turn dialog evaluation, and longitudinal outcome-oriented validation.

From a healthcare implementation perspective, the evaluated chatbot systems should be regarded as information-generating tools rather than clinically validated decision-support or patient-education interventions. Although the present study assessed their responses against guideline-based evidence and patient-information quality instruments, it did not validate these systems in real patients, clinical workflows, or outcome-based settings. Therefore, the findings support cautious, supervised use for educational assistance only, and further prospective validation involving patients, clinicians, comprehension testing, and safety-related monitoring is required before integration into routine heart failure education.

Although several chatbot systems generated generally high-quality patient education content, hallucinated responses were identified in all evaluated systems. Current LLMs should therefore be considered supportive educational tools rather than substitutes for professional medical consultation. Integration of LLM-generated information into HF education workflows should include clinician oversight, guideline-based verification, and explicit safeguards for high-risk topics, particularly medication management, symptom escalation, device therapy, and procedural decision-making (23, 24). Future patient-facing deployment should also incorporate transparent source attribution, uncertainty disclosure, emergency red-flag warnings, and clear escalation pathways for questions requiring individualized clinical judgment.

## 5 Limitations

This study has several limitations. First, the findings should therefore be interpreted as a time-specific evaluation rather than a permanent ranking of chatbot performance. Given the rapid evolution of LLM-based chatbot systems, subsequent model updates, interface changes, and modifications to retrieval or safety policies may alter their performance. The findings should therefore be interpreted as a time-specific evaluation, and future studies should incorporate longitudinal testing and explicit version tracking.

Second, although all systems were evaluated using a standardized protocol reported with reference to emerging guidance for chatbot health advice studies (21), the platforms differed in their retrieval capabilities. ChatGPT and DeepSeek were tested with web browsing disabled or unavailable, whereas Perplexity and Copilot operated using their default online retrieval modes. These configurations reflect the user-facing conditions available at the time of testing but may have influenced response content, source use, factual reliability, response length, and readability. Consequently, the observed differences cannot be attributed solely to the underlying language models. In addition, the evaluated chatbot systems may have been affected by model-level biases arising from their training data, alignment procedures, safety policies, and, for retrieval-enabled systems, the sources prioritized during online search. Such biases may have influenced the framing, completeness, and emphasis of heart failure information, including the potential underrepresentation of certain populations, healthcare systems, cultural contexts, or non-English patient-education needs. Because the internal training data, model-updating procedures, and retrieval-ranking mechanisms were not accessible, this study could not determine the extent to which the observed differences reflected model architecture, source selection, platform-specific safety filtering, or underlying data bias.

Third, the single-turn, context-isolated testing design did not fully reproduce real-world patient-chatbot interactions. In practice, users may provide personal clinical information, ask follow-up questions, seek clarification, or modify their questions in response to previous outputs. Future evaluations should therefore include multi-turn and scenario-based interactions, particularly for clinically sensitive topics such as medication use, worsening symptoms, device therapy, and thresholds for seeking urgent care.

Fourth, mDISCERN and EQIP primarily assess information quality, structure, completeness, and patient-oriented presentation rather than factual accuracy or direct clinical safety outcomes. Although all 432 responses underwent a response-level hallucination audit against the 2022 AHA/ACC/HFSA Heart Failure Guideline and other relevant clinical evidence, the classification process still required expert clinical judgment. Therefore, the hallucination audit should be interpreted as an assessment of potential safety risks associated with misinformation rather than a direct evaluation of clinical safety. This was particularly relevant for context-dependent recommendations, areas of evolving evidence, and statements not directly addressed in the reference guideline. Although adjudication procedures and inter-rater agreement were used to improve consistency, some degree of subjectivity could not be eliminated. In addition, the adapted 12-item mDISCERN instrument used in this study has not been independently validated specifically for LLM-generated heart failure information, which may limit direct comparability with studies using the original DISCERN instrument or other modified versions.

Fifth, hallucination events were relatively infrequent, which limited the precision of between-system comparisons. Although paired binary analyses, exact pairwise testing, and multiplicity adjustment were used, the resulting odds ratios and pairwise differences should be interpreted cautiously, particularly where confidence intervals were wide or adjusted comparisons were not statistically significant.

Sixth, each question was submitted only once to each chatbot system; therefore, the reproducibility and within-system variability of responses across repeated generations were not evaluated. In addition, all readability formulas were developed for English-language text and estimate linguistic complexity rather than actual patient comprehension. The study did not include patients, evaluate usability or behavioral responses, or measure effects on self-management, adherence, healthcare use, or clinical outcomes. Future research should incorporate repeated-response testing, patient comprehension and usability assessments, multilingual evaluations, and outcome-oriented studies conducted in real-world HF education settings.

## 6 Conclusion

This study identified differences in patient-information quality, readability, and response-level hallucination outcomes among four publicly accessible LLM-based chatbot systems in response to standardized heart failure-related questions. Under the tested access and retrieval conditions, DeepSeek generally showed the most favorable content-quality profile and the lowest observed hallucination rate. Paired binary analyses suggested significant between-system differences in hallucination outcomes, primarily driven by the higher hallucination rate observed for Perplexity. However, readability rankings varied across calculators, hallucination events were relatively infrequent, and hallucinated or potentially misleading responses were identified across all chatbot systems. These findings suggest that current LLM-based chatbot systems should not be used as unsupervised sources of patient-facing heart failure education.

## Data Availability

The original contributions presented in the study are included in the article/Supplementary material, further inquiries can be directed to the corresponding author.

## Supplementary material

SUPPLEMENTARY APPENDIX A Contains the complete list of 108 standardized HF questions.

SUPPLEMENTARY APPENDIX B Contains the scoring templates for mDISCERN and EQIP.

SUPPLEMENTARY TABLE S1 Provide chatbot access conditions, model identifiers, retrieval settings, and generation-setting documentation.

SUPPLEMENTARY TABLE S2 Provide chatbot access conditions, model identifiers, retrieval settings, and generation-setting documentation.

SUPPLEMENTARY TABLE S3 provide detailed readability results and Tukey-adjusted mixed-model pairwise comparisons.

SUPPLEMENTARY TABLE S4 Provide detailed readability results and Tukey-adjusted mixed-model pairwise comparisons.

SUPPLEMENTARY TABLE S5 Provides hallucination rates by model and question domain.

SUPPLEMENTARY TABLE S6 Provides the full 432-row response-level hallucination audit, including response-level hallucination classification, error type, potential severity, flagged statement excerpt, audit rationale, reference/evidence basis, and final adjudicated decision.

SUPPLEMENTARY TABLE S7 Provides paired binary hallucination analyses, including Cochran’s Q test, GEE logistic regression, and exact pairwise McNemar tests.

SUPPLEMENTARY TABLE S8 Provides inter-rater reliability and internal consistency results.

SUPPLEMENTARY FIGURE S1 Provides bar chart comparisons of secondary readability metrics across chatbot systems.
