# Supplementary material for: Comparative quality, readability, and factual reliability of four publicly accessible chatbot systems for heart failure patient education: a cross-sectional evaluation
Source: Front Public Health. 2026 Oct 15;14:1966612. doi: 10.3389/fpubh.2026.1966612 (PMC13716745; doi:10.3389/fpubh.2026.1966612)
Supplement: Supplementary file 1 [file Supplementary_file_1.docx]

**Supplementary Material**

**Comparative Quality, Readability, and Factual Reliability of Four Publicly Accessible Chatbot Systems for Heart Failure Patient Education: A Cross-Sectional Evaluation**

This supplementary file provides additional methodological details, model-access documentation, secondary readability analyses, response-level hallucination audit details, paired binary analyses, inter-rater reliability results, the full question set, and scoring templates. Tables and figures are numbered consecutively as Supplementary Tables S1-S8, Supplementary Figure S1, and Appendices A-B to match the revised main manuscript. The completed CHART checklist is submitted as a separate reporting checklist with manuscript section and page references. The response-level hallucination audit dataset, Tukey-adjusted pairwise comparison results, and complete raw chatbot responses are supplied as separate machine-readable CSV files.

# Supplementary methods and definitions

**Reference standard for factual reliability.** The hallucination audit used the 2022 AHA/ACC/HFSA Heart Failure Guideline as the primary reference standard. Relevant clinical evidence also included major contemporary heart failure guidelines and authoritative patient-education sources used during question-set refinement, including AHA heart-failure patient-education resources and the 2023 focused update of the ESC heart failure guideline. When a statement was not directly addressed by the 2022 AHA/ACC/HFSA guideline, reviewers assessed whether it was supported by consistent contemporary guideline-based or peer-reviewed clinical evidence. Statements were classified as hallucinated only when they were clinically meaningful and guideline-inconsistent, contradicted established clinical evidence, were unsupported by applicable evidence, were overgeneralized beyond the relevant patient population, or could mislead patient-facing heart failure education.

**Response-level classification.** Hallucination was assessed at the response level. A response was classified as containing a hallucination if it included at least one clinically meaningful statement meeting the definition above. Non-answer formatting, response length, and general readability did not determine hallucination status. All four chatbot systems answered the same 108 questions; therefore, the full audit retained all 432 response-level records.

**Potential severity definitions.** Low severity indicates an imprecise, unsupported, or overgeneralized statement that is unlikely to cause immediate harm but may confuse patients or reduce educational accuracy. Moderate severity indicates a statement that could plausibly influence medication use, supplement use, treatment expectations, care-seeking behavior, or self-management decisions if followed without clinician review. High severity indicates a statement that could reasonably encourage unsafe action, delay emergency care, or contradict core guideline-directed treatment. No high-severity hallucinations were identified in the audited responses.

**Statistical reporting.** Continuous outcomes were analyzed using linear mixed-effects models with chatbot system as a fixed effect and question ID as a random intercept. Pairwise comparisons used Tukey adjustment. Hallucination outcomes were analyzed using paired binary methods because the same 108 questions were answered by all four chatbot systems. Wilson 95% confidence intervals were calculated for hallucination proportions, Cochran’s Q test was used for the overall paired comparison, exact McNemar tests with multiplicity adjustment were used for pairwise binary comparisons, and GEE logistic regression was used to account for question-level clustering. Non-percentage numeric results are reported to three decimal places. Percentages and corresponding 95% confidence intervals are reported to two decimal places. Very small P values are reported as P < 0.001.

Supplementary Table S1. Model identifiers, access conditions, system context, and session design of the evaluated chatbot systems.

*This table summarizes the technical specifications and experimental access conditions of the four LLM-based chatbot systems evaluated in this study, including provider, publicly available model information, access tier, browsing/retrieval status, session-handling protocol, and operating environment. All models were accessed between 1 and 5 August 2025 under a standardized testing protocol.*

| **Parameter** | **ChatGPT** | **DeepSeek** | **Perplexity** | **Copilot** |
| --- | --- | --- | --- | --- |
| Provider | OpenAI | DeepSeek AI | Perplexity AI Inc. | Microsoft Corp. |
| Underlying LLM (version/identifier) | GPT-4o; exact internal build not disclosed by the web interface | DeepSeek-V3 as accessed in August 2025; exact internal build not disclosed | Online model as reported by platform documentation at the time of testing; exact internal build not disclosed | GPT-4 family model as documented in Microsoft Edge Copilot at the time of testing; exact internal build not disclosed |
| Access tier/interface | Paid tier via chat.openai.com | Public web access via deepseek.ai | Pro tier via web interface | Free Edge-integrated version |
| Browsing/retrieval status | Browsing disabled; no RAG sources enabled | External browsing not available in the tested interface | Online retrieval enabled by default | Online retrieval via Bing enabled by default |
| Session design and context control | New session for each question; context cleared before input | Single-turn sessions with manual context reset after each query | Each query executed in a fresh tab; no conversation history retained | A new Copilot conversation/session was initiated for each question, and the prior context was cleared before every input. |
| Operating environment | Windows 11 Pro (build 23H2), Google Chrome v126.0 | Windows 11 Pro (build 23H2), Google Chrome v126.0 | Windows 11 Pro (build 23H2), Google Chrome v126.0 | Windows 11 Pro (build 23H2), Microsoft Edge v127.0 |
| Default system message/instruction | Public ChatGPT web-interface system context as displayed/available at testing | Public DeepSeek web-interface system context as displayed/available at testing | Public Perplexity web-interface system context with browsing enabled | Public Copilot web-interface system context integrated with Bing |
| Cache/context control | No memory or prior conversation retained for the tested session | Context manually reset after each query | Browser cache cleared periodically; no previous conversation retained | No prior Copilot chat context was retained; a context-cleared session was used for each question. |
| Access date | Aug 1–3, 2025 | Aug 3, 2025 | Aug 4–5, 2025 | Aug 5, 2025 |

*Note. All platforms were accessed using the same workstation and physical network to minimize environmental variability. Because web-based chatbot interfaces differ in their disclosure of model build numbers, parameter counts, and retrieval settings, unavailable information is reported as “not disclosed.” Browsing/retrieval status reflects the default or accessible configuration at the time of testing.*

Supplementary Table S2. Available, intended, or platform-reported generation settings documented for transparency.

| **Parameter** | **Value or setting** | **Notes** |
| --- | --- | --- |
| Language | English (US) | All questions were submitted in English; no translation or multilingual prompts were used. |
| Tone style | Neutral, patient-education oriented | The same standardized patient-facing prompt was used for all models. |
| Temperature | Not user-configurable/not disclosed for most web interfaces; platform default used | No manual temperature control was available or verifiable across all evaluated web interfaces; default settings were retained. |
| Top-p (nucleus sampling) | Not user-configurable/not disclosed for most web interfaces; platform default used | No manual top-p control was available or verifiable across all evaluated web interfaces; default settings were retained. |
| Maximum output length | Not uniformly configurable; platform default or available maximum used | The standardized prompt requested concise, organized, patient-facing responses. Output length settings were not uniformly available across platforms. |
| Frequency penalty | Not user-configurable/not disclosed for most web interfaces; platform default used | Non-configurable parameters were retained at platform defaults. |
| Presence penalty | Not user-configurable/not disclosed for most web interfaces; platform default used | Non-configurable parameters were retained at platform defaults. |

*Note. Because web-based chatbot interfaces do not permit manual configuration or verification of all sampling parameters, non-configurable settings were retained at platform defaults. These entries document accessible, intended, default, or platform-reported settings where available and are provided for transparency rather than to imply full experimental control across all platforms.*

Supplementary Table S3A. Calculator 1 secondary readability indices and Tukey-adjusted pairwise comparison summary.

| **Category** | **Metric** | **ChatGPT Mean ± SD** | **DeepSeek Mean ± SD** | **Perplexity Mean ± SD** | **Copilot Mean ± SD** | **Mixed-model P value** |
| --- | --- | --- | --- | --- | --- | --- |
| Basic knowledge (n = 50) | ARI | 10.370 ± 2.535ᵃᵇ | 12.324 ± 2.262ᵈᵉ | 16.267 ± 2.811ᶠ | 10.871 ± 3.140 | <0.001 |
| Basic knowledge (n = 50) | FRES | 45.520 ± 17.225ᵇ | 40.680 ± 13.425ᵈᵉ | 34.500 ± 13.737ᶠ | 47.440 ± 14.577 | <0.001 |
| Basic knowledge (n = 50) | GFOG | 11.362 ± 2.710ᵇ | 11.982 ± 1.820ᵈ | 13.284 ± 2.411ᶠ | 11.234 ± 2.146 | <0.001 |
| Basic knowledge (n = 50) | FKGL | 9.297 ± 2.508ᵇ | 9.973 ± 2.070ᵈ | 13.866 ± 2.414ᶠ | 9.396 ± 2.641 | <0.001 |
| Management (n = 41) | ARI | 10.996 ± 1.463ᵃᵇ | 13.722 ± 1.843ᵉ | 13.408 ± 2.334ᶠ | 11.983 ± 3.391 | <0.001 |
| Management (n = 41) | FRES | 45.415 ± 10.954ᵇ | 50.366 ± 13.074ᵈᵉ | 30.146 ± 14.161ᶠ | 43.390 ± 13.295 | <0.001 |
| Management (n = 41) | GFOG | 11.661 ± 1.700ᵇ | 11.644 ± 1.829ᵈ | 13.173 ± 2.349ᶠ | 12.254 ± 2.472 | <0.001 |
| Management (n = 41) | FKGL | 9.700 ± 1.506ᵇ | 8.855 ± 2.588ᵈᵉ | 12.036 ± 2.196ᶠ | 10.435 ± 2.780 | <0.001 |
| Other HF-related issues (n = 17) | ARI | 10.625 ± 1.445ᵃᵇ | 13.189 ± 1.736ᵉ | 12.394 ± 2.054 | 11.169 ± 1.462 | <0.001 |
| Other HF-related issues (n = 17) | FRES | 47.118 ± 8.957ᵃᵇ | 56.471 ± 13.063ᵈᵉ | 37.294 ± 13.499 | 44.176 ± 8.855 | <0.001 |
| Other HF-related issues (n = 17) | GFOG | 11.218 ± 1.674 | 11.229 ± 1.648 | 11.859 ± 2.248 | 11.900 ± 1.479 | 0.319 |
| Other HF-related issues (n = 17) | FKGL | 9.140 ± 1.395ᵃᵇ | 7.584 ± 2.535ᵈᵉ | 10.775 ± 1.836 | 9.733 ± 1.331 | <0.001 |

Note. Data are presented as mean ± SD and reported to three decimal places. ARI, Automated Readability Index; FRES, Flesch Reading Ease Score; GFOG, Gunning Fog Index; FKGL, Flesch-Kincaid Grade Level. Overall P values are from linear mixed-effects models with chatbot system as a fixed effect and question ID as a random intercept. Superscript letters indicate statistically significant Tukey-adjusted pairwise comparisons: a, ChatGPT vs DeepSeek; b, ChatGPT vs Perplexity; c, ChatGPT vs Copilot; d, DeepSeek vs Perplexity; e, DeepSeek vs Copilot; f, Perplexity vs Copilot. The expanded S3B table below lists all six pairwise comparisons for each domain and metric. Calculator 1: http://readabilityformulas.com/ .

S3B. Tukey-adjusted pairwise comparison summary for Calculator 1 secondary readability indices.

| **Category** | **Metric** | **Comparison** | **Observed mean difference** | **Observed direction** | **Tukey-adjusted significant** |
| --- | --- | --- | --- | --- | --- |
| Basic knowledge (n = 50) | ARI | ChatGPT vs DeepSeek | -1.954 | DeepSeek higher than ChatGPT | Yes |
| Basic knowledge (n = 50) | ARI | ChatGPT vs Perplexity | -5.897 | Perplexity higher than ChatGPT | Yes |
| Basic knowledge (n = 50) | ARI | ChatGPT vs Copilot | -0.501 | Copilot higher than ChatGPT | No |
| Basic knowledge (n = 50) | ARI | DeepSeek vs Perplexity | -3.943 | Perplexity higher than DeepSeek | Yes |
| Basic knowledge (n = 50) | ARI | DeepSeek vs Copilot | 1.453 | DeepSeek higher than Copilot | Yes |
| Basic knowledge (n = 50) | ARI | Perplexity vs Copilot | 5.396 | Perplexity higher than Copilot | Yes |
| Basic knowledge (n = 50) | FRES | ChatGPT vs DeepSeek | 4.840 | ChatGPT higher than DeepSeek | No |
| Basic knowledge (n = 50) | FRES | ChatGPT vs Perplexity | 11.020 | ChatGPT higher than Perplexity | Yes |
| Basic knowledge (n = 50) | FRES | ChatGPT vs Copilot | -1.920 | Copilot higher than ChatGPT | No |
| Basic knowledge (n = 50) | FRES | DeepSeek vs Perplexity | 6.180 | DeepSeek higher than Perplexity | Yes |
| Basic knowledge (n = 50) | FRES | DeepSeek vs Copilot | -6.760 | Copilot higher than DeepSeek | Yes |
| Basic knowledge (n = 50) | FRES | Perplexity vs Copilot | -12.940 | Copilot higher than Perplexity | Yes |
| Basic knowledge (n = 50) | GFOG | ChatGPT vs DeepSeek | -0.620 | DeepSeek higher than ChatGPT | No |
| Basic knowledge (n = 50) | GFOG | ChatGPT vs Perplexity | -1.922 | Perplexity higher than ChatGPT | Yes |
| Basic knowledge (n = 50) | GFOG | ChatGPT vs Copilot | 0.128 | ChatGPT higher than Copilot | No |
| Basic knowledge (n = 50) | GFOG | DeepSeek vs Perplexity | -1.302 | Perplexity higher than DeepSeek | Yes |
| Basic knowledge (n = 50) | GFOG | DeepSeek vs Copilot | 0.748 | DeepSeek higher than Copilot | No |
| Basic knowledge (n = 50) | GFOG | Perplexity vs Copilot | 2.050 | Perplexity higher than Copilot | Yes |
| Basic knowledge (n = 50) | FKGL | ChatGPT vs DeepSeek | -0.676 | DeepSeek higher than ChatGPT | No |
| Basic knowledge (n = 50) | FKGL | ChatGPT vs Perplexity | -4.569 | Perplexity higher than ChatGPT | Yes |
| Basic knowledge (n = 50) | FKGL | ChatGPT vs Copilot | -0.099 | Copilot higher than ChatGPT | No |
| Basic knowledge (n = 50) | FKGL | DeepSeek vs Perplexity | -3.893 | Perplexity higher than DeepSeek | Yes |
| Basic knowledge (n = 50) | FKGL | DeepSeek vs Copilot | 0.577 | DeepSeek higher than Copilot | No |
| Basic knowledge (n = 50) | FKGL | Perplexity vs Copilot | 4.470 | Perplexity higher than Copilot | Yes |
| Management (n = 41) | ARI | ChatGPT vs DeepSeek | -2.726 | DeepSeek higher than ChatGPT | Yes |
| Management (n = 41) | ARI | ChatGPT vs Perplexity | -2.412 | Perplexity higher than ChatGPT | Yes |
| Management (n = 41) | ARI | ChatGPT vs Copilot | -0.987 | Copilot higher than ChatGPT | No |
| Management (n = 41) | ARI | DeepSeek vs Perplexity | 0.314 | DeepSeek higher than Perplexity | No |
| Management (n = 41) | ARI | DeepSeek vs Copilot | 1.739 | DeepSeek higher than Copilot | Yes |
| Management (n = 41) | ARI | Perplexity vs Copilot | 1.425 | Perplexity higher than Copilot | Yes |
| Management (n = 41) | FRES | ChatGPT vs DeepSeek | -4.951 | DeepSeek higher than ChatGPT | No |
| Management (n = 41) | FRES | ChatGPT vs Perplexity | 15.269 | ChatGPT higher than Perplexity | Yes |
| Management (n = 41) | FRES | ChatGPT vs Copilot | 2.025 | ChatGPT higher than Copilot | No |
| Management (n = 41) | FRES | DeepSeek vs Perplexity | 20.220 | DeepSeek higher than Perplexity | Yes |
| Management (n = 41) | FRES | DeepSeek vs Copilot | 6.976 | DeepSeek higher than Copilot | Yes |
| Management (n = 41) | FRES | Perplexity vs Copilot | -13.244 | Copilot higher than Perplexity | Yes |
| Management (n = 41) | GFOG | ChatGPT vs DeepSeek | 0.017 | ChatGPT higher than DeepSeek | No |
| Management (n = 41) | GFOG | ChatGPT vs Perplexity | -1.512 | Perplexity higher than ChatGPT | Yes |
| Management (n = 41) | GFOG | ChatGPT vs Copilot | -0.593 | Copilot higher than ChatGPT | No |
| Management (n = 41) | GFOG | DeepSeek vs Perplexity | -1.529 | Perplexity higher than DeepSeek | Yes |
| Management (n = 41) | GFOG | DeepSeek vs Copilot | -0.610 | Copilot higher than DeepSeek | No |
| Management (n = 41) | GFOG | Perplexity vs Copilot | 0.919 | Perplexity higher than Copilot | Yes |
| Management (n = 41) | FKGL | ChatGPT vs DeepSeek | 0.845 | ChatGPT higher than DeepSeek | No |
| Management (n = 41) | FKGL | ChatGPT vs Perplexity | -2.336 | Perplexity higher than ChatGPT | Yes |
| Management (n = 41) | FKGL | ChatGPT vs Copilot | -0.735 | Copilot higher than ChatGPT | No |
| Management (n = 41) | FKGL | DeepSeek vs Perplexity | -3.181 | Perplexity higher than DeepSeek | Yes |
| Management (n = 41) | FKGL | DeepSeek vs Copilot | -1.580 | Copilot higher than DeepSeek | Yes |
| Management (n = 41) | FKGL | Perplexity vs Copilot | 1.601 | Perplexity higher than Copilot | Yes |
| Other HF-related issues (n = 17) | ARI | ChatGPT vs DeepSeek | -2.564 | DeepSeek higher than ChatGPT | Yes |
| Other HF-related issues (n = 17) | ARI | ChatGPT vs Perplexity | -1.769 | Perplexity higher than ChatGPT | Yes |
| Other HF-related issues (n = 17) | ARI | ChatGPT vs Copilot | -0.544 | Copilot higher than ChatGPT | No |
| Other HF-related issues (n = 17) | ARI | DeepSeek vs Perplexity | 0.795 | DeepSeek higher than Perplexity | No |
| Other HF-related issues (n = 17) | ARI | DeepSeek vs Copilot | 2.020 | DeepSeek higher than Copilot | Yes |
| Other HF-related issues (n = 17) | ARI | Perplexity vs Copilot | 1.225 | Perplexity higher than Copilot | No |
| Other HF-related issues (n = 17) | FRES | ChatGPT vs DeepSeek | -9.353 | DeepSeek higher than ChatGPT | Yes |
| Other HF-related issues (n = 17) | FRES | ChatGPT vs Perplexity | 9.824 | ChatGPT higher than Perplexity | Yes |
| Other HF-related issues (n = 17) | FRES | ChatGPT vs Copilot | 2.942 | ChatGPT higher than Copilot | No |
| Other HF-related issues (n = 17) | FRES | DeepSeek vs Perplexity | 19.177 | DeepSeek higher than Perplexity | Yes |
| Other HF-related issues (n = 17) | FRES | DeepSeek vs Copilot | 12.295 | DeepSeek higher than Copilot | Yes |
| Other HF-related issues (n = 17) | FRES | Perplexity vs Copilot | -6.882 | Copilot higher than Perplexity | No |
| Other HF-related issues (n = 17) | GFOG | ChatGPT vs DeepSeek | -0.011 | DeepSeek higher than ChatGPT | No |
| Other HF-related issues (n = 17) | GFOG | ChatGPT vs Perplexity | -0.641 | Perplexity higher than ChatGPT | No |
| Other HF-related issues (n = 17) | GFOG | ChatGPT vs Copilot | -0.682 | Copilot higher than ChatGPT | No |
| Other HF-related issues (n = 17) | GFOG | DeepSeek vs Perplexity | -0.630 | Perplexity higher than DeepSeek | No |
| Other HF-related issues (n = 17) | GFOG | DeepSeek vs Copilot | -0.671 | Copilot higher than DeepSeek | No |
| Other HF-related issues (n = 17) | GFOG | Perplexity vs Copilot | -0.041 | Copilot higher than Perplexity | No |
| Other HF-related issues (n = 17) | FKGL | ChatGPT vs DeepSeek | 1.556 | ChatGPT higher than DeepSeek | Yes |
| Other HF-related issues (n = 17) | FKGL | ChatGPT vs Perplexity | -1.635 | Perplexity higher than ChatGPT | Yes |
| Other HF-related issues (n = 17) | FKGL | ChatGPT vs Copilot | -0.593 | Copilot higher than ChatGPT | No |
| Other HF-related issues (n = 17) | FKGL | DeepSeek vs Perplexity | -3.191 | Perplexity higher than DeepSeek | Yes |
| Other HF-related issues (n = 17) | FKGL | DeepSeek vs Copilot | -2.149 | Copilot higher than DeepSeek | Yes |
| Other HF-related issues (n = 17) | FKGL | Perplexity vs Copilot | 1.042 | Perplexity higher than Copilot | No |

Note. Observed mean difference is calculated as the first-named model minus the second-named model using the summary means shown in S3A. Tukey-adjusted significance is derived from the superscript pairwise-comparison markers in S3A. Exact adjusted P values are supplied in the accompanying machine-readable pairwise-comparison file.

Supplementary Table S4A. Calculator 2 secondary readability indices and Tukey-adjusted pairwise comparison summary for sensitivity analysis.

| **Category** | **Metric** | **ChatGPT Mean ± SD** | **DeepSeek Mean ± SD** | **Perplexity Mean ± SD** | **Copilot Mean ± SD** | **Mixed-model P value** |
| --- | --- | --- | --- | --- | --- | --- |
| Basic knowledge (n = 50) | CLI | 14.671 ± 2.605 | 14.344 ± 2.113ᵈ | 15.426 ± 2.279ᶠ | 14.563 ± 2.345 | 0.003 |
| Basic knowledge (n = 50) | FKGL | 16.174 ± 13.619ᵃ | 10.868 ± 1.785ᵉ | 14.553 ± 2.316 | 17.646 ± 9.921 | <0.001 |
| Basic knowledge (n = 50) | ARI | 17.190 ± 17.519ᵃ | 10.481 ± 2.262ᵈᵉ | 15.777 ± 2.953 | 19.603 ± 12.744 | <0.001 |
| Management (n = 41) | CLI | 13.231 ± 1.780ᵇᶜ | 12.730 ± 2.069ᵈᵉ | 16.648 ± 1.940ᶠ | 14.412 ± 1.809 | <0.001 |
| Management (n = 41) | FKGL | 11.857 ± 2.860ᵃᶜ | 10.460 ± 1.611ᵉ | 10.885 ± 1.891ᶠ | 13.798 ± 3.651 | <0.001 |
| Management (n = 41) | ARI | 11.444 ± 3.121ᵃᶜ | 9.131 ± 2.154ᵈᵉ | 11.863 ± 2.047ᶠ | 14.042 ± 4.269 | <0.001 |
| Other HF-related issues (n = 17) | CLI | 13.534 ± 1.618ᵇᶜ | 12.455 ± 1.861ᵈᵉ | 16.416 ± 2.035 | 15.388 ± 1.760 | <0.001 |
| Other HF-related issues (n = 17) | FKGL | 10.272 ± 1.631ᶜ | 10.163 ± 1.515ᵉ | 10.501 ± 1.632ᶠ | 13.288 ± 2.585 | <0.001 |
| Other HF-related issues (n = 17) | ARI | 9.869 ± 1.511ᵇᶜ | 8.969 ± 1.810ᵈᵉ | 11.505 ± 1.714ᶠ | 13.805 ± 2.958 | <0.001 |

Note. Data are presented as mean ± SD and reported to three decimal places. CLI, Coleman-Liau Index; FKGL, Flesch-Kincaid Grade Level; ARI, Automated Readability Index. Overall P values are from linear mixed-effects models with chatbot system as a fixed effect and question ID as a random intercept. Superscript letters indicate statistically significant Tukey-adjusted pairwise comparisons: a, ChatGPT vs DeepSeek; b, ChatGPT vs Perplexity; c, ChatGPT vs Copilot; d, DeepSeek vs Perplexity; e, DeepSeek vs Copilot; f, Perplexity vs Copilot. The expanded S4B table below lists all six pairwise comparisons for each domain and metric. Calculator 2: https://www.online-utility.org/english/readability_test_and_improve.jsp.

S4B. Tukey-adjusted pairwise comparison summary for Calculator 2 secondary readability indices.

| **Category** | **Metric** | **Comparison** | **Observed mean difference** | **Observed direction** | **Tukey-adjusted significant** |
| --- | --- | --- | --- | --- | --- |
| Basic knowledge (n = 50) | CLI | ChatGPT vs DeepSeek | 0.327 | ChatGPT higher than DeepSeek | No |
| Basic knowledge (n = 50) | CLI | ChatGPT vs Perplexity | -0.755 | Perplexity higher than ChatGPT | No |
| Basic knowledge (n = 50) | CLI | ChatGPT vs Copilot | 0.108 | ChatGPT higher than Copilot | No |
| Basic knowledge (n = 50) | CLI | DeepSeek vs Perplexity | -1.082 | Perplexity higher than DeepSeek | Yes |
| Basic knowledge (n = 50) | CLI | DeepSeek vs Copilot | -0.219 | Copilot higher than DeepSeek | No |
| Basic knowledge (n = 50) | CLI | Perplexity vs Copilot | 0.863 | Perplexity higher than Copilot | Yes |
| Basic knowledge (n = 50) | FKGL | ChatGPT vs DeepSeek | 5.306 | ChatGPT higher than DeepSeek | Yes |
| Basic knowledge (n = 50) | FKGL | ChatGPT vs Perplexity | 1.621 | ChatGPT higher than Perplexity | No |
| Basic knowledge (n = 50) | FKGL | ChatGPT vs Copilot | -1.472 | Copilot higher than ChatGPT | No |
| Basic knowledge (n = 50) | FKGL | DeepSeek vs Perplexity | -3.685 | Perplexity higher than DeepSeek | No |
| Basic knowledge (n = 50) | FKGL | DeepSeek vs Copilot | -6.778 | Copilot higher than DeepSeek | Yes |
| Basic knowledge (n = 50) | FKGL | Perplexity vs Copilot | -3.093 | Copilot higher than Perplexity | No |
| Basic knowledge (n = 50) | ARI | ChatGPT vs DeepSeek | 6.709 | ChatGPT higher than DeepSeek | Yes |
| Basic knowledge (n = 50) | ARI | ChatGPT vs Perplexity | 1.413 | ChatGPT higher than Perplexity | No |
| Basic knowledge (n = 50) | ARI | ChatGPT vs Copilot | -2.413 | Copilot higher than ChatGPT | No |
| Basic knowledge (n = 50) | ARI | DeepSeek vs Perplexity | -5.296 | Perplexity higher than DeepSeek | Yes |
| Basic knowledge (n = 50) | ARI | DeepSeek vs Copilot | -9.122 | Copilot higher than DeepSeek | Yes |
| Basic knowledge (n = 50) | ARI | Perplexity vs Copilot | -3.826 | Copilot higher than Perplexity | No |
| Management (n = 41) | CLI | ChatGPT vs DeepSeek | 0.501 | ChatGPT higher than DeepSeek | No |
| Management (n = 41) | CLI | ChatGPT vs Perplexity | -3.417 | Perplexity higher than ChatGPT | Yes |
| Management (n = 41) | CLI | ChatGPT vs Copilot | -1.181 | Copilot higher than ChatGPT | Yes |
| Management (n = 41) | CLI | DeepSeek vs Perplexity | -3.918 | Perplexity higher than DeepSeek | Yes |
| Management (n = 41) | CLI | DeepSeek vs Copilot | -1.682 | Copilot higher than DeepSeek | Yes |
| Management (n = 41) | CLI | Perplexity vs Copilot | 2.236 | Perplexity higher than Copilot | Yes |
| Management (n = 41) | FKGL | ChatGPT vs DeepSeek | 1.397 | ChatGPT higher than DeepSeek | Yes |
| Management (n = 41) | FKGL | ChatGPT vs Perplexity | 0.972 | ChatGPT higher than Perplexity | No |
| Management (n = 41) | FKGL | ChatGPT vs Copilot | -1.941 | Copilot higher than ChatGPT | Yes |
| Management (n = 41) | FKGL | DeepSeek vs Perplexity | -0.425 | Perplexity higher than DeepSeek | No |
| Management (n = 41) | FKGL | DeepSeek vs Copilot | -3.338 | Copilot higher than DeepSeek | Yes |
| Management (n = 41) | FKGL | Perplexity vs Copilot | -2.913 | Copilot higher than Perplexity | Yes |
| Management (n = 41) | ARI | ChatGPT vs DeepSeek | 2.313 | ChatGPT higher than DeepSeek | Yes |
| Management (n = 41) | ARI | ChatGPT vs Perplexity | -0.419 | Perplexity higher than ChatGPT | No |
| Management (n = 41) | ARI | ChatGPT vs Copilot | -2.598 | Copilot higher than ChatGPT | Yes |
| Management (n = 41) | ARI | DeepSeek vs Perplexity | -2.732 | Perplexity higher than DeepSeek | Yes |
| Management (n = 41) | ARI | DeepSeek vs Copilot | -4.911 | Copilot higher than DeepSeek | Yes |
| Management (n = 41) | ARI | Perplexity vs Copilot | -2.179 | Copilot higher than Perplexity | Yes |
| Other HF-related issues (n = 17) | CLI | ChatGPT vs DeepSeek | 1.079 | ChatGPT higher than DeepSeek | No |
| Other HF-related issues (n = 17) | CLI | ChatGPT vs Perplexity | -2.882 | Perplexity higher than ChatGPT | Yes |
| Other HF-related issues (n = 17) | CLI | ChatGPT vs Copilot | -1.854 | Copilot higher than ChatGPT | Yes |
| Other HF-related issues (n = 17) | CLI | DeepSeek vs Perplexity | -3.961 | Perplexity higher than DeepSeek | Yes |
| Other HF-related issues (n = 17) | CLI | DeepSeek vs Copilot | -2.933 | Copilot higher than DeepSeek | Yes |
| Other HF-related issues (n = 17) | CLI | Perplexity vs Copilot | 1.028 | Perplexity higher than Copilot | No |
| Other HF-related issues (n = 17) | FKGL | ChatGPT vs DeepSeek | 0.109 | ChatGPT higher than DeepSeek | No |
| Other HF-related issues (n = 17) | FKGL | ChatGPT vs Perplexity | -0.229 | Perplexity higher than ChatGPT | No |
| Other HF-related issues (n = 17) | FKGL | ChatGPT vs Copilot | -3.016 | Copilot higher than ChatGPT | Yes |
| Other HF-related issues (n = 17) | FKGL | DeepSeek vs Perplexity | -0.338 | Perplexity higher than DeepSeek | No |
| Other HF-related issues (n = 17) | FKGL | DeepSeek vs Copilot | -3.125 | Copilot higher than DeepSeek | Yes |
| Other HF-related issues (n = 17) | FKGL | Perplexity vs Copilot | -2.787 | Copilot higher than Perplexity | Yes |
| Other HF-related issues (n = 17) | ARI | ChatGPT vs DeepSeek | 0.900 | ChatGPT higher than DeepSeek | No |
| Other HF-related issues (n = 17) | ARI | ChatGPT vs Perplexity | -1.636 | Perplexity higher than ChatGPT | Yes |
| Other HF-related issues (n = 17) | ARI | ChatGPT vs Copilot | -3.936 | Copilot higher than ChatGPT | Yes |
| Other HF-related issues (n = 17) | ARI | DeepSeek vs Perplexity | -2.536 | Perplexity higher than DeepSeek | Yes |
| Other HF-related issues (n = 17) | ARI | DeepSeek vs Copilot | -4.836 | Copilot higher than DeepSeek | Yes |
| Other HF-related issues (n = 17) | ARI | Perplexity vs Copilot | -2.300 | Copilot higher than Perplexity | Yes |

Note. Observed mean difference is calculated as the first-named model minus the second-named model using the summary means shown in S4A. Tukey-adjusted significance is derived from the superscript pairwise-comparison markers in S4A. Exact adjusted P values are supplied in the accompanying machine-readable pairwise-comparison file.

Supplementary Table S5. Summary of the response-level hallucination audit by model and question domain.

| **Model** | **Basic knowledge n/N** | **Management n/N** | **Other n/N** | **Total hallucination n/N** | **Hallucination rate (%)** | **Wilson 95% CI** |
| --- | --- | --- | --- | --- | --- | --- |
| DeepSeek | 0/50 | 5/41 | 1/17 | 6/108 | 5.56 | 2.57-11.59 |
| ChatGPT | 1/50 | 6/41 | 1/17 | 8/108 | 7.41 | 3.80-13.94 |
| Copilot | 2/50 | 6/41 | 2/17 | 10/108 | 9.26 | 5.11-16.21 |
| Perplexity | 3/50 | 10/41 | 3/17 | 16/108 | 14.81 | 9.33-22.72 |

*Note. All 432 generated responses were audited at the response level. Hallucination rate was calculated as the proportion of responses containing at least one guideline-inconsistent, clinically unsupported, overgeneralized, or potentially misleading statement among all responses generated by each chatbot system. Hallucination percentages and Wilson 95% confidence intervals are reported to two decimal places.*

Supplementary Table S6. Full 432-row response-level hallucination audit dataset.

Supplementary Table S6 provides the full 432-row response-level hallucination audit dataset, including hallucination classification, error type, potential severity, flagged statement excerpt, audit rationale, reference/evidence basis, and final adjudicated decision. The machine-readable version is provided as a separate CSV file. The verbatim raw chatbot responses corresponding to all 432 model-question outputs are supplied separately as Supplementary Table S9.

| **Question ID** | **Category** | **Model** | **Hallucination** | **Error type** | **Potential severity** | **Flagged statement excerpt** | **Audit rationale** | **Reference standard/evidence basis** | **Final adjudicated decision** |
| --- | --- | --- | --- | --- | --- | --- | --- | --- | --- |
| 1 | Basic knowledge | ChatGPT | 0 | None identified | None | Not applicable | No clinically meaningful hallucination identified after audit. | Not applicable. | No hallucination identified. |
| 1 | Basic knowledge | DeepSeek | 0 | None identified | None | Not applicable | No clinically meaningful hallucination identified after audit. | Not applicable. | No hallucination identified. |
| 1 | Basic knowledge | Perplexity | 1 | Overstated organ-complication claim | Low | Liver damage potentially leading to liver failure; poor circulation and blood clots increase ischemic stroke risk | Overgeneralizes severe organ outcomes and stroke risk as direct HF consequences. | 2022 AHA/ACC/HFSA HF Guideline, HF definition/staging and comorbidity discussion: hepatic congestion and thromboembolic events can occur in selected contexts, but the guideline does not support liver failure or ischemic stroke as direct universal consequences of HF. | Hallucination identified, low potential severity. |
| 1 | Basic knowledge | Copilot | 0 | None identified | None | Not applicable | No clinically meaningful hallucination identified after audit. | Not applicable. | No hallucination identified. |
| 2 | Basic knowledge | ChatGPT | 0 | None identified | None | Not applicable | No clinically meaningful hallucination identified after audit. | Not applicable. | No hallucination identified. |
| 2 | Basic knowledge | DeepSeek | 0 | None identified | None | Not applicable | No clinically meaningful hallucination identified after audit. | Not applicable. | No hallucination identified. |
| 2 | Basic knowledge | Perplexity | 0 | None identified | None | Not applicable | No clinically meaningful hallucination identified after audit. | Not applicable. | No hallucination identified. |
| 2 | Basic knowledge | Copilot | 0 | None identified | None | Not applicable | No clinically meaningful hallucination identified after audit. | Not applicable. | No hallucination identified. |
| 3 | Basic knowledge | ChatGPT | 0 | None identified | None | Not applicable | No clinically meaningful hallucination identified after audit. | Not applicable. | No hallucination identified. |
| 3 | Basic knowledge | DeepSeek | 0 | None identified | None | Not applicable | No clinically meaningful hallucination identified after audit. | Not applicable. | No hallucination identified. |
| 3 | Basic knowledge | Perplexity | 0 | None identified | None | Not applicable | No clinically meaningful hallucination identified after audit. | Not applicable. | No hallucination identified. |
| 3 | Basic knowledge | Copilot | 0 | None identified | None | Not applicable | No clinically meaningful hallucination identified after audit. | Not applicable. | No hallucination identified. |
| 4 | Basic knowledge | ChatGPT | 0 | None identified | None | Not applicable | No clinically meaningful hallucination identified after audit. | Not applicable. | No hallucination identified. |
| 4 | Basic knowledge | DeepSeek | 0 | None identified | None | Not applicable | No clinically meaningful hallucination identified after audit. | Not applicable. | No hallucination identified. |
| 4 | Basic knowledge | Perplexity | 0 | None identified | None | Not applicable | No clinically meaningful hallucination identified after audit. | Not applicable. | No hallucination identified. |
| 4 | Basic knowledge | Copilot | 0 | None identified | None | Not applicable | No clinically meaningful hallucination identified after audit. | Not applicable. | No hallucination identified. |
| 5 | Basic knowledge | ChatGPT | 0 | None identified | None | Not applicable | No clinically meaningful hallucination identified after audit. | Not applicable. | No hallucination identified. |
| 5 | Basic knowledge | DeepSeek | 0 | None identified | None | Not applicable | No clinically meaningful hallucination identified after audit. | Not applicable. | No hallucination identified. |
| 5 | Basic knowledge | Perplexity | 0 | None identified | None | Not applicable | No clinically meaningful hallucination identified after audit. | Not applicable. | No hallucination identified. |
| 5 | Basic knowledge | Copilot | 1 | Unsupported pediatric prognosis statistics | Low | Only ~20% of CHD children develop HF; CHD-related HF 1-year survival ~82.5%; DCM 50% death/transplant in 5 years | Highly specific prognosis statistics are presented without context and are not guideline-based. | 2022 AHA/ACC/HFSA HF Guideline and contemporary pediatric HF/CHD literature context: the adult HF guideline does not support the exact pediatric CHD/DCM prognosis statistics stated; such disease-specific estimates require age-, diagnosis-, and cohort-specific pediatric evidence. | Hallucination identified, low potential severity. |
| 6 | Basic knowledge | ChatGPT | 0 | None identified | None | Not applicable | No clinically meaningful hallucination identified after audit. | Not applicable. | No hallucination identified. |
| 6 | Basic knowledge | DeepSeek | 0 | None identified | None | Not applicable | No clinically meaningful hallucination identified after audit. | Not applicable. | No hallucination identified. |
| 6 | Basic knowledge | Perplexity | 0 | None identified | None | Not applicable | No clinically meaningful hallucination identified after audit. | Not applicable. | No hallucination identified. |
| 6 | Basic knowledge | Copilot | 1 | Overstated viral myocarditis claim | Low | Parvovirus B19 is described as fatal myocarditis in children | Overstates a possible viral association as a generally fatal pediatric condition. | 2022 AHA/ACC/HFSA HF Guideline, myocarditis/etiology discussion and contemporary myocarditis evidence: viral myocarditis is a possible cause of HF, but the guideline does not support presenting parvovirus B19 as generally causing fatal myocarditis in children. | Hallucination identified, low potential severity. |
| 7 | Basic knowledge | ChatGPT | 0 | None identified | None | Not applicable | No clinically meaningful hallucination identified after audit. | Not applicable. | No hallucination identified. |
| 7 | Basic knowledge | DeepSeek | 0 | None identified | None | Not applicable | No clinically meaningful hallucination identified after audit. | Not applicable. | No hallucination identified. |
| 7 | Basic knowledge | Perplexity | 0 | None identified | None | Not applicable | No clinically meaningful hallucination identified after audit. | Not applicable. | No hallucination identified. |
| 7 | Basic knowledge | Copilot | 0 | None identified | None | Not applicable | No clinically meaningful hallucination identified after audit. | Not applicable. | No hallucination identified. |
| 8 | Basic knowledge | ChatGPT | 0 | None identified | None | Not applicable | No clinically meaningful hallucination identified after audit. | Not applicable. | No hallucination identified. |
| 8 | Basic knowledge | DeepSeek | 0 | None identified | None | Not applicable | No clinically meaningful hallucination identified after audit. | Not applicable. | No hallucination identified. |
| 8 | Basic knowledge | Perplexity | 1 | Unsupported numerical familial-risk claim | Low | Offspring of a parent with HF have a 70% higher risk | Specific risk estimate is not guideline-based and lacks context. | 2022 AHA/ACC/HFSA HF Guideline, genetic cardiomyopathy and family-screening recommendations: family history can justify screening in selected cardiomyopathies, but no guideline-based 70% offspring-risk estimate is provided for HF in general. | Hallucination identified, low potential severity. |
| 8 | Basic knowledge | Copilot | 0 | None identified | None | Not applicable | No clinically meaningful hallucination identified after audit. | Not applicable. | No hallucination identified. |
| 9 | Basic knowledge | ChatGPT | 0 | None identified | None | Not applicable | No clinically meaningful hallucination identified after audit. | Not applicable. | No hallucination identified. |
| 9 | Basic knowledge | DeepSeek | 0 | None identified | None | Not applicable | No clinically meaningful hallucination identified after audit. | Not applicable. | No hallucination identified. |
| 9 | Basic knowledge | Perplexity | 0 | None identified | None | Not applicable | No clinically meaningful hallucination identified after audit. | Not applicable. | No hallucination identified. |
| 9 | Basic knowledge | Copilot | 0 | None identified | None | Not applicable | No clinically meaningful hallucination identified after audit. | Not applicable. | No hallucination identified. |
| 10 | Basic knowledge | ChatGPT | 0 | None identified | None | Not applicable | No clinically meaningful hallucination identified after audit. | Not applicable. | No hallucination identified. |
| 10 | Basic knowledge | DeepSeek | 0 | None identified | None | Not applicable | No clinically meaningful hallucination identified after audit. | Not applicable. | No hallucination identified. |
| 10 | Basic knowledge | Perplexity | 0 | None identified | None | Not applicable | No clinically meaningful hallucination identified after audit. | Not applicable. | No hallucination identified. |
| 10 | Basic knowledge | Copilot | 0 | None identified | None | Not applicable | No clinically meaningful hallucination identified after audit. | Not applicable. | No hallucination identified. |
| 11 | Basic knowledge | ChatGPT | 0 | None identified | None | Not applicable | No clinically meaningful hallucination identified after audit. | Not applicable. | No hallucination identified. |
| 11 | Basic knowledge | DeepSeek | 0 | None identified | None | Not applicable | No clinically meaningful hallucination identified after audit. | Not applicable. | No hallucination identified. |
| 11 | Basic knowledge | Perplexity | 0 | None identified | None | Not applicable | No clinically meaningful hallucination identified after audit. | Not applicable. | No hallucination identified. |
| 11 | Basic knowledge | Copilot | 0 | None identified | None | Not applicable | No clinically meaningful hallucination identified after audit. | Not applicable. | No hallucination identified. |
| 12 | Basic knowledge | ChatGPT | 0 | None identified | None | Not applicable | No clinically meaningful hallucination identified after audit. | Not applicable. | No hallucination identified. |
| 12 | Basic knowledge | DeepSeek | 0 | None identified | None | Not applicable | No clinically meaningful hallucination identified after audit. | Not applicable. | No hallucination identified. |
| 12 | Basic knowledge | Perplexity | 0 | None identified | None | Not applicable | No clinically meaningful hallucination identified after audit. | Not applicable. | No hallucination identified. |
| 12 | Basic knowledge | Copilot | 0 | None identified | None | Not applicable | No clinically meaningful hallucination identified after audit. | Not applicable. | No hallucination identified. |
| 13 | Basic knowledge | ChatGPT | 0 | None identified | None | Not applicable | No clinically meaningful hallucination identified after audit. | Not applicable. | No hallucination identified. |
| 13 | Basic knowledge | DeepSeek | 0 | None identified | None | Not applicable | No clinically meaningful hallucination identified after audit. | Not applicable. | No hallucination identified. |
| 13 | Basic knowledge | Perplexity | 0 | None identified | None | Not applicable | No clinically meaningful hallucination identified after audit. | Not applicable. | No hallucination identified. |
| 13 | Basic knowledge | Copilot | 0 | None identified | None | Not applicable | No clinically meaningful hallucination identified after audit. | Not applicable. | No hallucination identified. |
| 14 | Basic knowledge | ChatGPT | 0 | None identified | None | Not applicable | No clinically meaningful hallucination identified after audit. | Not applicable. | No hallucination identified. |
| 14 | Basic knowledge | DeepSeek | 0 | None identified | None | Not applicable | No clinically meaningful hallucination identified after audit. | Not applicable. | No hallucination identified. |
| 14 | Basic knowledge | Perplexity | 0 | None identified | None | Not applicable | No clinically meaningful hallucination identified after audit. | Not applicable. | No hallucination identified. |
| 14 | Basic knowledge | Copilot | 0 | None identified | None | Not applicable | No clinically meaningful hallucination identified after audit. | Not applicable. | No hallucination identified. |
| 15 | Basic knowledge | ChatGPT | 0 | None identified | None | Not applicable | No clinically meaningful hallucination identified after audit. | Not applicable. | No hallucination identified. |
| 15 | Basic knowledge | DeepSeek | 0 | None identified | None | Not applicable | No clinically meaningful hallucination identified after audit. | Not applicable. | No hallucination identified. |
| 15 | Basic knowledge | Perplexity | 0 | None identified | None | Not applicable | No clinically meaningful hallucination identified after audit. | Not applicable. | No hallucination identified. |
| 15 | Basic knowledge | Copilot | 0 | None identified | None | Not applicable | No clinically meaningful hallucination identified after audit. | Not applicable. | No hallucination identified. |
| 16 | Basic knowledge | ChatGPT | 1 | Unsupported epidemiologic/causal claim | Low | Heart failure reported in 10–25% of hospitalized COVID-19 patients; direct viral invasion via ACE2 | Overstates/quantifies COVID-related HF and presents debated mechanism as established. | 2022 AHA/ACC/HFSA HF Guideline and contemporary COVID-19 cardiovascular literature: COVID-19 may be associated with myocardial injury or HF in selected cases, but fixed hospitalization percentages and direct ACE2-mediated chronic HF mechanisms should not be presented as established general HF facts. | Hallucination identified, low potential severity. |
| 16 | Basic knowledge | DeepSeek | 0 | None identified | None | Not applicable | No clinically meaningful hallucination identified after audit. | Not applicable. | No hallucination identified. |
| 16 | Basic knowledge | Perplexity | 1 | Unsupported chronic viral persistence claim | Low | Persistent or reactivated viral infections may contribute to chronic heart damage | Too broad for patient-facing HF education without qualification. | 2022 AHA/ACC/HFSA HF Guideline and contemporary COVID-19 cardiovascular literature: COVID-19 may be associated with myocardial injury or HF in selected cases, but fixed hospitalization percentages and direct ACE2-mediated chronic HF mechanisms should not be presented as established general HF facts. | Hallucination identified, low potential severity. |
| 16 | Basic knowledge | Copilot | 0 | None identified | None | Not applicable | No clinically meaningful hallucination identified after audit. | Not applicable. | No hallucination identified. |
| 17 | Basic knowledge | ChatGPT | 0 | None identified | None | Not applicable | No clinically meaningful hallucination identified after audit. | Not applicable. | No hallucination identified. |
| 17 | Basic knowledge | DeepSeek | 0 | None identified | None | Not applicable | No clinically meaningful hallucination identified after audit. | Not applicable. | No hallucination identified. |
| 17 | Basic knowledge | Perplexity | 0 | None identified | None | Not applicable | No clinically meaningful hallucination identified after audit. | Not applicable. | No hallucination identified. |
| 17 | Basic knowledge | Copilot | 0 | None identified | None | Not applicable | No clinically meaningful hallucination identified after audit. | Not applicable. | No hallucination identified. |
| 18 | Basic knowledge | ChatGPT | 0 | None identified | None | Not applicable | No clinically meaningful hallucination identified after audit. | Not applicable. | No hallucination identified. |
| 18 | Basic knowledge | DeepSeek | 0 | None identified | None | Not applicable | No clinically meaningful hallucination identified after audit. | Not applicable. | No hallucination identified. |
| 18 | Basic knowledge | Perplexity | 0 | None identified | None | Not applicable | No clinically meaningful hallucination identified after audit. | Not applicable. | No hallucination identified. |
| 18 | Basic knowledge | Copilot | 0 | None identified | None | Not applicable | No clinically meaningful hallucination identified after audit. | Not applicable. | No hallucination identified. |
| 19 | Basic knowledge | ChatGPT | 0 | None identified | None | Not applicable | No clinically meaningful hallucination identified after audit. | Not applicable. | No hallucination identified. |
| 19 | Basic knowledge | DeepSeek | 0 | None identified | None | Not applicable | No clinically meaningful hallucination identified after audit. | Not applicable. | No hallucination identified. |
| 19 | Basic knowledge | Perplexity | 0 | None identified | None | Not applicable | No clinically meaningful hallucination identified after audit. | Not applicable. | No hallucination identified. |
| 19 | Basic knowledge | Copilot | 0 | None identified | None | Not applicable | No clinically meaningful hallucination identified after audit. | Not applicable. | No hallucination identified. |
| 20 | Basic knowledge | ChatGPT | 0 | None identified | None | Not applicable | No clinically meaningful hallucination identified after audit. | Not applicable. | No hallucination identified. |
| 20 | Basic knowledge | DeepSeek | 0 | None identified | None | Not applicable | No clinically meaningful hallucination identified after audit. | Not applicable. | No hallucination identified. |
| 20 | Basic knowledge | Perplexity | 0 | None identified | None | Not applicable | No clinically meaningful hallucination identified after audit. | Not applicable. | No hallucination identified. |
| 20 | Basic knowledge | Copilot | 0 | None identified | None | Not applicable | No clinically meaningful hallucination identified after audit. | Not applicable. | No hallucination identified. |
| 21 | Basic knowledge | ChatGPT | 0 | None identified | None | Not applicable | No clinically meaningful hallucination identified after audit. | Not applicable. | No hallucination identified. |
| 21 | Basic knowledge | DeepSeek | 0 | None identified | None | Not applicable | No clinically meaningful hallucination identified after audit. | Not applicable. | No hallucination identified. |
| 21 | Basic knowledge | Perplexity | 0 | None identified | None | Not applicable | No clinically meaningful hallucination identified after audit. | Not applicable. | No hallucination identified. |
| 21 | Basic knowledge | Copilot | 0 | None identified | None | Not applicable | No clinically meaningful hallucination identified after audit. | Not applicable. | No hallucination identified. |
| 22 | Basic knowledge | ChatGPT | 0 | None identified | None | Not applicable | No clinically meaningful hallucination identified after audit. | Not applicable. | No hallucination identified. |
| 22 | Basic knowledge | DeepSeek | 0 | None identified | None | Not applicable | No clinically meaningful hallucination identified after audit. | Not applicable. | No hallucination identified. |
| 22 | Basic knowledge | Perplexity | 0 | None identified | None | Not applicable | No clinically meaningful hallucination identified after audit. | Not applicable. | No hallucination identified. |
| 22 | Basic knowledge | Copilot | 0 | None identified | None | Not applicable | No clinically meaningful hallucination identified after audit. | Not applicable. | No hallucination identified. |
| 23 | Basic knowledge | ChatGPT | 0 | None identified | None | Not applicable | No clinically meaningful hallucination identified after audit. | Not applicable. | No hallucination identified. |
| 23 | Basic knowledge | DeepSeek | 0 | None identified | None | Not applicable | No clinically meaningful hallucination identified after audit. | Not applicable. | No hallucination identified. |
| 23 | Basic knowledge | Perplexity | 0 | None identified | None | Not applicable | No clinically meaningful hallucination identified after audit. | Not applicable. | No hallucination identified. |
| 23 | Basic knowledge | Copilot | 0 | None identified | None | Not applicable | No clinically meaningful hallucination identified after audit. | Not applicable. | No hallucination identified. |
| 24 | Basic knowledge | ChatGPT | 0 | None identified | None | Not applicable | No clinically meaningful hallucination identified after audit. | Not applicable. | No hallucination identified. |
| 24 | Basic knowledge | DeepSeek | 0 | None identified | None | Not applicable | No clinically meaningful hallucination identified after audit. | Not applicable. | No hallucination identified. |
| 24 | Basic knowledge | Perplexity | 0 | None identified | None | Not applicable | No clinically meaningful hallucination identified after audit. | Not applicable. | No hallucination identified. |
| 24 | Basic knowledge | Copilot | 0 | None identified | None | Not applicable | No clinically meaningful hallucination identified after audit. | Not applicable. | No hallucination identified. |
| 25 | Basic knowledge | ChatGPT | 0 | None identified | None | Not applicable | No clinically meaningful hallucination identified after audit. | Not applicable. | No hallucination identified. |
| 25 | Basic knowledge | DeepSeek | 0 | None identified | None | Not applicable | No clinically meaningful hallucination identified after audit. | Not applicable. | No hallucination identified. |
| 25 | Basic knowledge | Perplexity | 0 | None identified | None | Not applicable | No clinically meaningful hallucination identified after audit. | Not applicable. | No hallucination identified. |
| 25 | Basic knowledge | Copilot | 0 | None identified | None | Not applicable | No clinically meaningful hallucination identified after audit. | Not applicable. | No hallucination identified. |
| 26 | Basic knowledge | ChatGPT | 0 | None identified | None | Not applicable | No clinically meaningful hallucination identified after audit. | Not applicable. | No hallucination identified. |
| 26 | Basic knowledge | DeepSeek | 0 | None identified | None | Not applicable | No clinically meaningful hallucination identified after audit. | Not applicable. | No hallucination identified. |
| 26 | Basic knowledge | Perplexity | 0 | None identified | None | Not applicable | No clinically meaningful hallucination identified after audit. | Not applicable. | No hallucination identified. |
| 26 | Basic knowledge | Copilot | 0 | None identified | None | Not applicable | No clinically meaningful hallucination identified after audit. | Not applicable. | No hallucination identified. |
| 27 | Basic knowledge | ChatGPT | 0 | None identified | None | Not applicable | No clinically meaningful hallucination identified after audit. | Not applicable. | No hallucination identified. |
| 27 | Basic knowledge | DeepSeek | 0 | None identified | None | Not applicable | No clinically meaningful hallucination identified after audit. | Not applicable. | No hallucination identified. |
| 27 | Basic knowledge | Perplexity | 0 | None identified | None | Not applicable | No clinically meaningful hallucination identified after audit. | Not applicable. | No hallucination identified. |
| 27 | Basic knowledge | Copilot | 0 | None identified | None | Not applicable | No clinically meaningful hallucination identified after audit. | Not applicable. | No hallucination identified. |
| 28 | Basic knowledge | ChatGPT | 0 | None identified | None | Not applicable | No clinically meaningful hallucination identified after audit. | Not applicable. | No hallucination identified. |
| 28 | Basic knowledge | DeepSeek | 0 | None identified | None | Not applicable | No clinically meaningful hallucination identified after audit. | Not applicable. | No hallucination identified. |
| 28 | Basic knowledge | Perplexity | 0 | None identified | None | Not applicable | No clinically meaningful hallucination identified after audit. | Not applicable. | No hallucination identified. |
| 28 | Basic knowledge | Copilot | 0 | None identified | None | Not applicable | No clinically meaningful hallucination identified after audit. | Not applicable. | No hallucination identified. |
| 29 | Basic knowledge | ChatGPT | 0 | None identified | None | Not applicable | No clinically meaningful hallucination identified after audit. | Not applicable. | No hallucination identified. |
| 29 | Basic knowledge | DeepSeek | 0 | None identified | None | Not applicable | No clinically meaningful hallucination identified after audit. | Not applicable. | No hallucination identified. |
| 29 | Basic knowledge | Perplexity | 0 | None identified | None | Not applicable | No clinically meaningful hallucination identified after audit. | Not applicable. | No hallucination identified. |
| 29 | Basic knowledge | Copilot | 0 | None identified | None | Not applicable | No clinically meaningful hallucination identified after audit. | Not applicable. | No hallucination identified. |
| 30 | Basic knowledge | ChatGPT | 0 | None identified | None | Not applicable | No clinically meaningful hallucination identified after audit. | Not applicable. | No hallucination identified. |
| 30 | Basic knowledge | DeepSeek | 0 | None identified | None | Not applicable | No clinically meaningful hallucination identified after audit. | Not applicable. | No hallucination identified. |
| 30 | Basic knowledge | Perplexity | 0 | None identified | None | Not applicable | No clinically meaningful hallucination identified after audit. | Not applicable. | No hallucination identified. |
| 30 | Basic knowledge | Copilot | 0 | None identified | None | Not applicable | No clinically meaningful hallucination identified after audit. | Not applicable. | No hallucination identified. |
| 31 | Basic knowledge | ChatGPT | 0 | None identified | None | Not applicable | No clinically meaningful hallucination identified after audit. | Not applicable. | No hallucination identified. |
| 31 | Basic knowledge | DeepSeek | 0 | None identified | None | Not applicable | No clinically meaningful hallucination identified after audit. | Not applicable. | No hallucination identified. |
| 31 | Basic knowledge | Perplexity | 0 | None identified | None | Not applicable | No clinically meaningful hallucination identified after audit. | Not applicable. | No hallucination identified. |
| 31 | Basic knowledge | Copilot | 0 | None identified | None | Not applicable | No clinically meaningful hallucination identified after audit. | Not applicable. | No hallucination identified. |
| 32 | Basic knowledge | ChatGPT | 0 | None identified | None | Not applicable | No clinically meaningful hallucination identified after audit. | Not applicable. | No hallucination identified. |
| 32 | Basic knowledge | DeepSeek | 0 | None identified | None | Not applicable | No clinically meaningful hallucination identified after audit. | Not applicable. | No hallucination identified. |
| 32 | Basic knowledge | Perplexity | 0 | None identified | None | Not applicable | No clinically meaningful hallucination identified after audit. | Not applicable. | No hallucination identified. |
| 32 | Basic knowledge | Copilot | 0 | None identified | None | Not applicable | No clinically meaningful hallucination identified after audit. | Not applicable. | No hallucination identified. |
| 33 | Basic knowledge | ChatGPT | 0 | None identified | None | Not applicable | No clinically meaningful hallucination identified after audit. | Not applicable. | No hallucination identified. |
| 33 | Basic knowledge | DeepSeek | 0 | None identified | None | Not applicable | No clinically meaningful hallucination identified after audit. | Not applicable. | No hallucination identified. |
| 33 | Basic knowledge | Perplexity | 0 | None identified | None | Not applicable | No clinically meaningful hallucination identified after audit. | Not applicable. | No hallucination identified. |
| 33 | Basic knowledge | Copilot | 0 | None identified | None | Not applicable | No clinically meaningful hallucination identified after audit. | Not applicable. | No hallucination identified. |
| 34 | Basic knowledge | ChatGPT | 0 | None identified | None | Not applicable | No clinically meaningful hallucination identified after audit. | Not applicable. | No hallucination identified. |
| 34 | Basic knowledge | DeepSeek | 0 | None identified | None | Not applicable | No clinically meaningful hallucination identified after audit. | Not applicable. | No hallucination identified. |
| 34 | Basic knowledge | Perplexity | 0 | None identified | None | Not applicable | No clinically meaningful hallucination identified after audit. | Not applicable. | No hallucination identified. |
| 34 | Basic knowledge | Copilot | 0 | None identified | None | Not applicable | No clinically meaningful hallucination identified after audit. | Not applicable. | No hallucination identified. |
| 35 | Basic knowledge | ChatGPT | 0 | None identified | None | Not applicable | No clinically meaningful hallucination identified after audit. | Not applicable. | No hallucination identified. |
| 35 | Basic knowledge | DeepSeek | 0 | None identified | None | Not applicable | No clinically meaningful hallucination identified after audit. | Not applicable. | No hallucination identified. |
| 35 | Basic knowledge | Perplexity | 0 | None identified | None | Not applicable | No clinically meaningful hallucination identified after audit. | Not applicable. | No hallucination identified. |
| 35 | Basic knowledge | Copilot | 0 | None identified | None | Not applicable | No clinically meaningful hallucination identified after audit. | Not applicable. | No hallucination identified. |
| 36 | Basic knowledge | ChatGPT | 0 | None identified | None | Not applicable | No clinically meaningful hallucination identified after audit. | Not applicable. | No hallucination identified. |
| 36 | Basic knowledge | DeepSeek | 0 | None identified | None | Not applicable | No clinically meaningful hallucination identified after audit. | Not applicable. | No hallucination identified. |
| 36 | Basic knowledge | Perplexity | 0 | None identified | None | Not applicable | No clinically meaningful hallucination identified after audit. | Not applicable. | No hallucination identified. |
| 36 | Basic knowledge | Copilot | 0 | None identified | None | Not applicable | No clinically meaningful hallucination identified after audit. | Not applicable. | No hallucination identified. |
| 37 | Basic knowledge | ChatGPT | 0 | None identified | None | Not applicable | No clinically meaningful hallucination identified after audit. | Not applicable. | No hallucination identified. |
| 37 | Basic knowledge | DeepSeek | 0 | None identified | None | Not applicable | No clinically meaningful hallucination identified after audit. | Not applicable. | No hallucination identified. |
| 37 | Basic knowledge | Perplexity | 0 | None identified | None | Not applicable | No clinically meaningful hallucination identified after audit. | Not applicable. | No hallucination identified. |
| 37 | Basic knowledge | Copilot | 0 | None identified | None | Not applicable | No clinically meaningful hallucination identified after audit. | Not applicable. | No hallucination identified. |
| 38 | Basic knowledge | ChatGPT | 0 | None identified | None | Not applicable | No clinically meaningful hallucination identified after audit. | Not applicable. | No hallucination identified. |
| 38 | Basic knowledge | DeepSeek | 0 | None identified | None | Not applicable | No clinically meaningful hallucination identified after audit. | Not applicable. | No hallucination identified. |
| 38 | Basic knowledge | Perplexity | 0 | None identified | None | Not applicable | No clinically meaningful hallucination identified after audit. | Not applicable. | No hallucination identified. |
| 38 | Basic knowledge | Copilot | 0 | None identified | None | Not applicable | No clinically meaningful hallucination identified after audit. | Not applicable. | No hallucination identified. |
| 39 | Basic knowledge | ChatGPT | 0 | None identified | None | Not applicable | No clinically meaningful hallucination identified after audit. | Not applicable. | No hallucination identified. |
| 39 | Basic knowledge | DeepSeek | 0 | None identified | None | Not applicable | No clinically meaningful hallucination identified after audit. | Not applicable. | No hallucination identified. |
| 39 | Basic knowledge | Perplexity | 0 | None identified | None | Not applicable | No clinically meaningful hallucination identified after audit. | Not applicable. | No hallucination identified. |
| 39 | Basic knowledge | Copilot | 0 | None identified | None | Not applicable | No clinically meaningful hallucination identified after audit. | Not applicable. | No hallucination identified. |
| 40 | Basic knowledge | ChatGPT | 0 | None identified | None | Not applicable | No clinically meaningful hallucination identified after audit. | Not applicable. | No hallucination identified. |
| 40 | Basic knowledge | DeepSeek | 0 | None identified | None | Not applicable | No clinically meaningful hallucination identified after audit. | Not applicable. | No hallucination identified. |
| 40 | Basic knowledge | Perplexity | 0 | None identified | None | Not applicable | No clinically meaningful hallucination identified after audit. | Not applicable. | No hallucination identified. |
| 40 | Basic knowledge | Copilot | 0 | None identified | None | Not applicable | No clinically meaningful hallucination identified after audit. | Not applicable. | No hallucination identified. |
| 41 | Basic knowledge | ChatGPT | 0 | None identified | None | Not applicable | No clinically meaningful hallucination identified after audit. | Not applicable. | No hallucination identified. |
| 41 | Basic knowledge | DeepSeek | 0 | None identified | None | Not applicable | No clinically meaningful hallucination identified after audit. | Not applicable. | No hallucination identified. |
| 41 | Basic knowledge | Perplexity | 0 | None identified | None | Not applicable | No clinically meaningful hallucination identified after audit. | Not applicable. | No hallucination identified. |
| 41 | Basic knowledge | Copilot | 0 | None identified | None | Not applicable | No clinically meaningful hallucination identified after audit. | Not applicable. | No hallucination identified. |
| 42 | Basic knowledge | ChatGPT | 0 | None identified | None | Not applicable | No clinically meaningful hallucination identified after audit. | Not applicable. | No hallucination identified. |
| 42 | Basic knowledge | DeepSeek | 0 | None identified | None | Not applicable | No clinically meaningful hallucination identified after audit. | Not applicable. | No hallucination identified. |
| 42 | Basic knowledge | Perplexity | 0 | None identified | None | Not applicable | No clinically meaningful hallucination identified after audit. | Not applicable. | No hallucination identified. |
| 42 | Basic knowledge | Copilot | 0 | None identified | None | Not applicable | No clinically meaningful hallucination identified after audit. | Not applicable. | No hallucination identified. |
| 43 | Basic knowledge | ChatGPT | 0 | None identified | None | Not applicable | No clinically meaningful hallucination identified after audit. | Not applicable. | No hallucination identified. |
| 43 | Basic knowledge | DeepSeek | 0 | None identified | None | Not applicable | No clinically meaningful hallucination identified after audit. | Not applicable. | No hallucination identified. |
| 43 | Basic knowledge | Perplexity | 0 | None identified | None | Not applicable | No clinically meaningful hallucination identified after audit. | Not applicable. | No hallucination identified. |
| 43 | Basic knowledge | Copilot | 0 | None identified | None | Not applicable | No clinically meaningful hallucination identified after audit. | Not applicable. | No hallucination identified. |
| 44 | Basic knowledge | ChatGPT | 0 | None identified | None | Not applicable | No clinically meaningful hallucination identified after audit. | Not applicable. | No hallucination identified. |
| 44 | Basic knowledge | DeepSeek | 0 | None identified | None | Not applicable | No clinically meaningful hallucination identified after audit. | Not applicable. | No hallucination identified. |
| 44 | Basic knowledge | Perplexity | 0 | None identified | None | Not applicable | No clinically meaningful hallucination identified after audit. | Not applicable. | No hallucination identified. |
| 44 | Basic knowledge | Copilot | 0 | None identified | None | Not applicable | No clinically meaningful hallucination identified after audit. | Not applicable. | No hallucination identified. |
| 45 | Basic knowledge | ChatGPT | 0 | None identified | None | Not applicable | No clinically meaningful hallucination identified after audit. | Not applicable. | No hallucination identified. |
| 45 | Basic knowledge | DeepSeek | 0 | None identified | None | Not applicable | No clinically meaningful hallucination identified after audit. | Not applicable. | No hallucination identified. |
| 45 | Basic knowledge | Perplexity | 0 | None identified | None | Not applicable | No clinically meaningful hallucination identified after audit. | Not applicable. | No hallucination identified. |
| 45 | Basic knowledge | Copilot | 0 | None identified | None | Not applicable | No clinically meaningful hallucination identified after audit. | Not applicable. | No hallucination identified. |
| 46 | Basic knowledge | ChatGPT | 0 | None identified | None | Not applicable | No clinically meaningful hallucination identified after audit. | Not applicable. | No hallucination identified. |
| 46 | Basic knowledge | DeepSeek | 0 | None identified | None | Not applicable | No clinically meaningful hallucination identified after audit. | Not applicable. | No hallucination identified. |
| 46 | Basic knowledge | Perplexity | 0 | None identified | None | Not applicable | No clinically meaningful hallucination identified after audit. | Not applicable. | No hallucination identified. |
| 46 | Basic knowledge | Copilot | 0 | None identified | None | Not applicable | No clinically meaningful hallucination identified after audit. | Not applicable. | No hallucination identified. |
| 47 | Basic knowledge | ChatGPT | 0 | None identified | None | Not applicable | No clinically meaningful hallucination identified after audit. | Not applicable. | No hallucination identified. |
| 47 | Basic knowledge | DeepSeek | 0 | None identified | None | Not applicable | No clinically meaningful hallucination identified after audit. | Not applicable. | No hallucination identified. |
| 47 | Basic knowledge | Perplexity | 0 | None identified | None | Not applicable | No clinically meaningful hallucination identified after audit. | Not applicable. | No hallucination identified. |
| 47 | Basic knowledge | Copilot | 0 | None identified | None | Not applicable | No clinically meaningful hallucination identified after audit. | Not applicable. | No hallucination identified. |
| 48 | Basic knowledge | ChatGPT | 0 | None identified | None | Not applicable | No clinically meaningful hallucination identified after audit. | Not applicable. | No hallucination identified. |
| 48 | Basic knowledge | DeepSeek | 0 | None identified | None | Not applicable | No clinically meaningful hallucination identified after audit. | Not applicable. | No hallucination identified. |
| 48 | Basic knowledge | Perplexity | 0 | None identified | None | Not applicable | No clinically meaningful hallucination identified after audit. | Not applicable. | No hallucination identified. |
| 48 | Basic knowledge | Copilot | 0 | None identified | None | Not applicable | No clinically meaningful hallucination identified after audit. | Not applicable. | No hallucination identified. |
| 49 | Basic knowledge | ChatGPT | 0 | None identified | None | Not applicable | No clinically meaningful hallucination identified after audit. | Not applicable. | No hallucination identified. |
| 49 | Basic knowledge | DeepSeek | 0 | None identified | None | Not applicable | No clinically meaningful hallucination identified after audit. | Not applicable. | No hallucination identified. |
| 49 | Basic knowledge | Perplexity | 0 | None identified | None | Not applicable | No clinically meaningful hallucination identified after audit. | Not applicable. | No hallucination identified. |
| 49 | Basic knowledge | Copilot | 0 | None identified | None | Not applicable | No clinically meaningful hallucination identified after audit. | Not applicable. | No hallucination identified. |
| 50 | Basic knowledge | ChatGPT | 0 | None identified | None | Not applicable | No clinically meaningful hallucination identified after audit. | Not applicable. | No hallucination identified. |
| 50 | Basic knowledge | DeepSeek | 0 | None identified | None | Not applicable | No clinically meaningful hallucination identified after audit. | Not applicable. | No hallucination identified. |
| 50 | Basic knowledge | Perplexity | 0 | None identified | None | Not applicable | No clinically meaningful hallucination identified after audit. | Not applicable. | No hallucination identified. |
| 50 | Basic knowledge | Copilot | 0 | None identified | None | Not applicable | No clinically meaningful hallucination identified after audit. | Not applicable. | No hallucination identified. |
| 51 | Management | ChatGPT | 0 | None identified | None | Not applicable | No clinically meaningful hallucination identified after audit. | Not applicable. | No hallucination identified. |
| 51 | Management | DeepSeek | 0 | None identified | None | Not applicable | No clinically meaningful hallucination identified after audit. | Not applicable. | No hallucination identified. |
| 51 | Management | Perplexity | 0 | None identified | None | Not applicable | No clinically meaningful hallucination identified after audit. | Not applicable. | No hallucination identified. |
| 51 | Management | Copilot | 0 | None identified | None | Not applicable | No clinically meaningful hallucination identified after audit. | Not applicable. | No hallucination identified. |
| 52 | Management | ChatGPT | 0 | None identified | None | Not applicable | No clinically meaningful hallucination identified after audit. | Not applicable. | No hallucination identified. |
| 52 | Management | DeepSeek | 0 | None identified | None | Not applicable | No clinically meaningful hallucination identified after audit. | Not applicable. | No hallucination identified. |
| 52 | Management | Perplexity | 0 | None identified | None | Not applicable | No clinically meaningful hallucination identified after audit. | Not applicable. | No hallucination identified. |
| 52 | Management | Copilot | 0 | None identified | None | Not applicable | No clinically meaningful hallucination identified after audit. | Not applicable. | No hallucination identified. |
| 53 | Management | ChatGPT | 0 | None identified | None | Not applicable | No clinically meaningful hallucination identified after audit. | Not applicable. | No hallucination identified. |
| 53 | Management | DeepSeek | 0 | None identified | None | Not applicable | No clinically meaningful hallucination identified after audit. | Not applicable. | No hallucination identified. |
| 53 | Management | Perplexity | 0 | None identified | None | Not applicable | No clinically meaningful hallucination identified after audit. | Not applicable. | No hallucination identified. |
| 53 | Management | Copilot | 0 | None identified | None | Not applicable | No clinically meaningful hallucination identified after audit. | Not applicable. | No hallucination identified. |
| 54 | Management | ChatGPT | 0 | None identified | None | Not applicable | No clinically meaningful hallucination identified after audit. | Not applicable. | No hallucination identified. |
| 54 | Management | DeepSeek | 0 | None identified | None | Not applicable | No clinically meaningful hallucination identified after audit. | Not applicable. | No hallucination identified. |
| 54 | Management | Perplexity | 0 | None identified | None | Not applicable | No clinically meaningful hallucination identified after audit. | Not applicable. | No hallucination identified. |
| 54 | Management | Copilot | 0 | None identified | None | Not applicable | No clinically meaningful hallucination identified after audit. | Not applicable. | No hallucination identified. |
| 55 | Management | ChatGPT | 0 | None identified | None | Not applicable | No clinically meaningful hallucination identified after audit. | Not applicable. | No hallucination identified. |
| 55 | Management | DeepSeek | 0 | None identified | None | Not applicable | No clinically meaningful hallucination identified after audit. | Not applicable. | No hallucination identified. |
| 55 | Management | Perplexity | 0 | None identified | None | Not applicable | No clinically meaningful hallucination identified after audit. | Not applicable. | No hallucination identified. |
| 55 | Management | Copilot | 0 | None identified | None | Not applicable | No clinically meaningful hallucination identified after audit. | Not applicable. | No hallucination identified. |
| 56 | Management | ChatGPT | 0 | None identified | None | Not applicable | No clinically meaningful hallucination identified after audit. | Not applicable. | No hallucination identified. |
| 56 | Management | DeepSeek | 0 | None identified | None | Not applicable | No clinically meaningful hallucination identified after audit. | Not applicable. | No hallucination identified. |
| 56 | Management | Perplexity | 0 | None identified | None | Not applicable | No clinically meaningful hallucination identified after audit. | Not applicable. | No hallucination identified. |
| 56 | Management | Copilot | 0 | None identified | None | Not applicable | No clinically meaningful hallucination identified after audit. | Not applicable. | No hallucination identified. |
| 57 | Management | ChatGPT | 0 | None identified | None | Not applicable | No clinically meaningful hallucination identified after audit. | Not applicable. | No hallucination identified. |
| 57 | Management | DeepSeek | 0 | None identified | None | Not applicable | No clinically meaningful hallucination identified after audit. | Not applicable. | No hallucination identified. |
| 57 | Management | Perplexity | 0 | None identified | None | Not applicable | No clinically meaningful hallucination identified after audit. | Not applicable. | No hallucination identified. |
| 57 | Management | Copilot | 0 | None identified | None | Not applicable | No clinically meaningful hallucination identified after audit. | Not applicable. | No hallucination identified. |
| 58 | Management | ChatGPT | 0 | None identified | None | Not applicable | No clinically meaningful hallucination identified after audit. | Not applicable. | No hallucination identified. |
| 58 | Management | DeepSeek | 0 | None identified | None | Not applicable | No clinically meaningful hallucination identified after audit. | Not applicable. | No hallucination identified. |
| 58 | Management | Perplexity | 0 | None identified | None | Not applicable | No clinically meaningful hallucination identified after audit. | Not applicable. | No hallucination identified. |
| 58 | Management | Copilot | 0 | None identified | None | Not applicable | No clinically meaningful hallucination identified after audit. | Not applicable. | No hallucination identified. |
| 59 | Management | ChatGPT | 0 | None identified | None | Not applicable | No clinically meaningful hallucination identified after audit. | Not applicable. | No hallucination identified. |
| 59 | Management | DeepSeek | 0 | None identified | None | Not applicable | No clinically meaningful hallucination identified after audit. | Not applicable. | No hallucination identified. |
| 59 | Management | Perplexity | 0 | None identified | None | Not applicable | No clinically meaningful hallucination identified after audit. | Not applicable. | No hallucination identified. |
| 59 | Management | Copilot | 0 | None identified | None | Not applicable | No clinically meaningful hallucination identified after audit. | Not applicable. | No hallucination identified. |
| 60 | Management | ChatGPT | 0 | None identified | None | Not applicable | No clinically meaningful hallucination identified after audit. | Not applicable. | No hallucination identified. |
| 60 | Management | DeepSeek | 0 | None identified | None | Not applicable | No clinically meaningful hallucination identified after audit. | Not applicable. | No hallucination identified. |
| 60 | Management | Perplexity | 0 | None identified | None | Not applicable | No clinically meaningful hallucination identified after audit. | Not applicable. | No hallucination identified. |
| 60 | Management | Copilot | 0 | None identified | None | Not applicable | No clinically meaningful hallucination identified after audit. | Not applicable. | No hallucination identified. |
| 61 | Management | ChatGPT | 0 | None identified | None | Not applicable | No clinically meaningful hallucination identified after audit. | Not applicable. | No hallucination identified. |
| 61 | Management | DeepSeek | 0 | None identified | None | Not applicable | No clinically meaningful hallucination identified after audit. | Not applicable. | No hallucination identified. |
| 61 | Management | Perplexity | 0 | None identified | None | Not applicable | No clinically meaningful hallucination identified after audit. | Not applicable. | No hallucination identified. |
| 61 | Management | Copilot | 0 | None identified | None | Not applicable | No clinically meaningful hallucination identified after audit. | Not applicable. | No hallucination identified. |
| 62 | Management | ChatGPT | 0 | None identified | None | Not applicable | No clinically meaningful hallucination identified after audit. | Not applicable. | No hallucination identified. |
| 62 | Management | DeepSeek | 0 | None identified | None | Not applicable | No clinically meaningful hallucination identified after audit. | Not applicable. | No hallucination identified. |
| 62 | Management | Perplexity | 0 | None identified | None | Not applicable | No clinically meaningful hallucination identified after audit. | Not applicable. | No hallucination identified. |
| 62 | Management | Copilot | 0 | None identified | None | Not applicable | No clinically meaningful hallucination identified after audit. | Not applicable. | No hallucination identified. |
| 63 | Management | ChatGPT | 0 | None identified | None | Not applicable | No clinically meaningful hallucination identified after audit. | Not applicable. | No hallucination identified. |
| 63 | Management | DeepSeek | 0 | None identified | None | Not applicable | No clinically meaningful hallucination identified after audit. | Not applicable. | No hallucination identified. |
| 63 | Management | Perplexity | 0 | None identified | None | Not applicable | No clinically meaningful hallucination identified after audit. | Not applicable. | No hallucination identified. |
| 63 | Management | Copilot | 0 | None identified | None | Not applicable | No clinically meaningful hallucination identified after audit. | Not applicable. | No hallucination identified. |
| 64 | Management | ChatGPT | 1 | Overly prescriptive sodium target / numeric imprecision | Low | Limit sodium to <2,000 mg/day, roughly about 1 teaspoon of salt | Universal fixed sodium limits are not strongly guideline-mandated; 1 tsp salt is approximately 2,300 mg sodium. | 2022 AHA/ACC/HFSA HF Guideline, patient self-care/dietary counseling recommendations: dietary sodium advice should be individualized; the guideline does not support a universal “non-negotiable” sodium target, a survival claim, or a fixed 30% hospitalization reduction from sodium restriction. | Hallucination identified, low potential severity. |
| 64 | Management | DeepSeek | 1 | Unsupported/overstated sodium benefit | Moderate | Strict sodium restriction is non-negotiable; improves survival; 1,000 mg/day reduction lowers hospitalizations by 30% | Universal strict sodium mandate and quantified survival/hospitalization benefit are not supported as stated. | 2022 AHA/ACC/HFSA HF Guideline, patient self-care/dietary counseling recommendations: dietary sodium advice should be individualized; the guideline does not support a universal “non-negotiable” sodium target, a survival claim, or a fixed 30% hospitalization reduction from sodium restriction. | Hallucination identified, moderate potential severity. |
| 64 | Management | Perplexity | 1 | Over-specific sodium ranges and benefit claim | Low | Recommended sodium intake 1,500–3,000 mg; reducing sodium can reduce hospitalizations | Fixed sodium ranges and hospitalization benefit are not established for all HF patients. | 2022 AHA/ACC/HFSA HF Guideline, patient self-care/dietary counseling recommendations: dietary sodium advice should be individualized; the guideline does not support a universal “non-negotiable” sodium target, a survival claim, or a fixed 30% hospitalization reduction from sodium restriction. | Hallucination identified, low potential severity. |
| 64 | Management | Copilot | 1 | Over-specific sodium target | Low | Recommended salt intake generally <1,500–2,000 mg/day | Fixed sodium targets are not uniformly guideline-mandated for all HF patients. | 2022 AHA/ACC/HFSA HF Guideline, patient self-care/dietary counseling recommendations: dietary sodium advice should be individualized; the guideline does not support a universal “non-negotiable” sodium target, a survival claim, or a fixed 30% hospitalization reduction from sodium restriction. | Hallucination identified, low potential severity. |
| 65 | Management | ChatGPT | 0 | None identified | None | Not applicable | No clinically meaningful hallucination identified after audit. | Not applicable. | No hallucination identified. |
| 65 | Management | DeepSeek | 1 | Overstated universal fluid restriction | Low | Fluid intake typically restricted to 1.5–2.0 L/day; restriction is critical | Fluid restriction is individualized and most relevant to selected patients with congestion/hyponatremia. | 2022 AHA/ACC/HFSA HF Guideline, congestion and self-care management: fluid restriction is context-dependent and generally reserved for selected patients such as those with congestion or hyponatremia; it is not a universal 1.5–2.0 L/day recommendation for all HF patients. | Hallucination identified, low potential severity. |
| 65 | Management | Perplexity | 1 | Overgeneralized fluid restriction | Low | Recommended fluid intake is generally 1.5–2.0 L/day | Fluid restriction should be individualized. | 2022 AHA/ACC/HFSA HF Guideline, congestion and self-care management: fluid restriction is context-dependent and generally reserved for selected patients such as those with congestion or hyponatremia; it is not a universal 1.5–2.0 L/day recommendation for all HF patients. | Hallucination identified, low potential severity. |
| 65 | Management | Copilot | 0 | None identified | None | Not applicable | No clinically meaningful hallucination identified after audit. | Not applicable. | No hallucination identified. |
| 66 | Management | ChatGPT | 0 | None identified | None | Not applicable | No clinically meaningful hallucination identified after audit. | Not applicable. | No hallucination identified. |
| 66 | Management | DeepSeek | 0 | None identified | None | Not applicable | No clinically meaningful hallucination identified after audit. | Not applicable. | No hallucination identified. |
| 66 | Management | Perplexity | 0 | None identified | None | Not applicable | No clinically meaningful hallucination identified after audit. | Not applicable. | No hallucination identified. |
| 66 | Management | Copilot | 0 | None identified | None | Not applicable | No clinically meaningful hallucination identified after audit. | Not applicable. | No hallucination identified. |
| 67 | Management | ChatGPT | 0 | None identified | None | Not applicable | No clinically meaningful hallucination identified after audit. | Not applicable. | No hallucination identified. |
| 67 | Management | DeepSeek | 0 | None identified | None | Not applicable | No clinically meaningful hallucination identified after audit. | Not applicable. | No hallucination identified. |
| 67 | Management | Perplexity | 0 | None identified | None | Not applicable | No clinically meaningful hallucination identified after audit. | Not applicable. | No hallucination identified. |
| 67 | Management | Copilot | 0 | None identified | None | Not applicable | No clinically meaningful hallucination identified after audit. | Not applicable. | No hallucination identified. |
| 68 | Management | ChatGPT | 0 | None identified | None | Not applicable | No clinically meaningful hallucination identified after audit. | Not applicable. | No hallucination identified. |
| 68 | Management | DeepSeek | 0 | None identified | None | Not applicable | No clinically meaningful hallucination identified after audit. | Not applicable. | No hallucination identified. |
| 68 | Management | Perplexity | 0 | None identified | None | Not applicable | No clinically meaningful hallucination identified after audit. | Not applicable. | No hallucination identified. |
| 68 | Management | Copilot | 0 | None identified | None | Not applicable | No clinically meaningful hallucination identified after audit. | Not applicable. | No hallucination identified. |
| 69 | Management | ChatGPT | 0 | None identified | None | Not applicable | No clinically meaningful hallucination identified after audit. | Not applicable. | No hallucination identified. |
| 69 | Management | DeepSeek | 0 | None identified | None | Not applicable | No clinically meaningful hallucination identified after audit. | Not applicable. | No hallucination identified. |
| 69 | Management | Perplexity | 0 | None identified | None | Not applicable | No clinically meaningful hallucination identified after audit. | Not applicable. | No hallucination identified. |
| 69 | Management | Copilot | 0 | None identified | None | Not applicable | No clinically meaningful hallucination identified after audit. | Not applicable. | No hallucination identified. |
| 70 | Management | ChatGPT | 1 | Non-HF-specific glycemic targets | Low | Fasting 80–130 mg/dL, postprandial <180 mg/dL, HbA1c <7% | These are general diabetes targets, not HF-specific targets; should be individualized. | 2022 AHA/ACC/HFSA HF Guideline and contemporary diabetes-care standards: SGLT2 inhibitors are guideline-directed for eligible HF patients, but HF guidelines do not define a single HF-specific fasting glucose or HbA1c target; glycemic targets require individualized diabetes management. | Hallucination identified, low potential severity. |
| 70 | Management | DeepSeek | 1 | Unsupported HF-specific glycemic targets | Low | HbA1c 7.0–8.0%, fasting glucose 100–180 mg/dL for most HF patients with diabetes | HF guidelines do not define a uniform glycemic target. | 2022 AHA/ACC/HFSA HF Guideline and contemporary diabetes-care standards: SGLT2 inhibitors are guideline-directed for eligible HF patients, but HF guidelines do not define a single HF-specific fasting glucose or HbA1c target; glycemic targets require individualized diabetes management. | Hallucination identified, low potential severity. |
| 70 | Management | Perplexity | 1 | Potentially unsafe glycemic target | Moderate | HbA1c around 7.5–9% may be preferable for HF patients with diabetes | Applies selected advanced-HF findings too broadly and may promote overly permissive glycemic control. | 2022 AHA/ACC/HFSA HF Guideline and contemporary diabetes-care standards: SGLT2 inhibitors are guideline-directed for eligible HF patients, but HF guidelines do not define a single HF-specific fasting glucose or HbA1c target; glycemic targets require individualized diabetes management. | Hallucination identified, moderate potential severity. |
| 70 | Management | Copilot | 1 | Unsupported HF-specific glycemic targets | Low | Most guidelines suggest specific glucose targets for HF patients with diabetes or prediabetes | No HF-specific glucose target is established; targets should be individualized. | 2022 AHA/ACC/HFSA HF Guideline and contemporary diabetes-care standards: SGLT2 inhibitors are guideline-directed for eligible HF patients, but HF guidelines do not define a single HF-specific fasting glucose or HbA1c target; glycemic targets require individualized diabetes management. | Hallucination identified, low potential severity. |
| 71 | Management | ChatGPT | 0 | None identified | None | Not applicable | No clinically meaningful hallucination identified after audit. | Not applicable. | No hallucination identified. |
| 71 | Management | DeepSeek | 0 | None identified | None | Not applicable | No clinically meaningful hallucination identified after audit. | Not applicable. | No hallucination identified. |
| 71 | Management | Perplexity | 1 | Overcompressed oxygen evidence | Low | Long-term oxygen did not improve symptoms or survival; not routinely recommended | Core conclusion is largely correct, but it may underemphasize individualized hypoxemia indications. | 2022 AHA/ACC/HFSA HF Guideline and contemporary HF evidence: oxygen therapy is indicated for hypoxemia or specific respiratory indications; routine long-term oxygen for non-hypoxemic HF patients is not established, and individualized hypoxemia assessment remains necessary. | Hallucination identified, low potential severity. |
| 71 | Management | Copilot | 0 | None identified | None | Not applicable | No clinically meaningful hallucination identified after audit. | Not applicable. | No hallucination identified. |
| 72 | Management | ChatGPT | 0 | None identified | None | Not applicable | No clinically meaningful hallucination identified after audit. | Not applicable. | No hallucination identified. |
| 72 | Management | DeepSeek | 0 | None identified | None | Not applicable | No clinically meaningful hallucination identified after audit. | Not applicable. | No hallucination identified. |
| 72 | Management | Perplexity | 0 | None identified | None | Not applicable | No clinically meaningful hallucination identified after audit. | Not applicable. | No hallucination identified. |
| 72 | Management | Copilot | 0 | None identified | None | Not applicable | No clinically meaningful hallucination identified after audit. | Not applicable. | No hallucination identified. |
| 73 | Management | ChatGPT | 0 | None identified | None | Not applicable | No clinically meaningful hallucination identified after audit. | Not applicable. | No hallucination identified. |
| 73 | Management | DeepSeek | 0 | None identified | None | Not applicable | No clinically meaningful hallucination identified after audit. | Not applicable. | No hallucination identified. |
| 73 | Management | Perplexity | 0 | None identified | None | Not applicable | No clinically meaningful hallucination identified after audit. | Not applicable. | No hallucination identified. |
| 73 | Management | Copilot | 0 | None identified | None | Not applicable | No clinically meaningful hallucination identified after audit. | Not applicable. | No hallucination identified. |
| 74 | Management | ChatGPT | 0 | None identified | None | Not applicable | No clinically meaningful hallucination identified after audit. | Not applicable. | No hallucination identified. |
| 74 | Management | DeepSeek | 0 | None identified | None | Not applicable | No clinically meaningful hallucination identified after audit. | Not applicable. | No hallucination identified. |
| 74 | Management | Perplexity | 0 | None identified | None | Not applicable | No clinically meaningful hallucination identified after audit. | Not applicable. | No hallucination identified. |
| 74 | Management | Copilot | 0 | None identified | None | Not applicable | No clinically meaningful hallucination identified after audit. | Not applicable. | No hallucination identified. |
| 75 | Management | ChatGPT | 0 | None identified | None | Not applicable | No clinically meaningful hallucination identified after audit. | Not applicable. | No hallucination identified. |
| 75 | Management | DeepSeek | 0 | None identified | None | Not applicable | No clinically meaningful hallucination identified after audit. | Not applicable. | No hallucination identified. |
| 75 | Management | Perplexity | 0 | None identified | None | Not applicable | No clinically meaningful hallucination identified after audit. | Not applicable. | No hallucination identified. |
| 75 | Management | Copilot | 0 | None identified | None | Not applicable | No clinically meaningful hallucination identified after audit. | Not applicable. | No hallucination identified. |
| 76 | Management | ChatGPT | 0 | None identified | None | Not applicable | No clinically meaningful hallucination identified after audit. | Not applicable. | No hallucination identified. |
| 76 | Management | DeepSeek | 0 | None identified | None | Not applicable | No clinically meaningful hallucination identified after audit. | Not applicable. | No hallucination identified. |
| 76 | Management | Perplexity | 0 | None identified | None | Not applicable | No clinically meaningful hallucination identified after audit. | Not applicable. | No hallucination identified. |
| 76 | Management | Copilot | 0 | None identified | None | Not applicable | No clinically meaningful hallucination identified after audit. | Not applicable. | No hallucination identified. |
| 77 | Management | ChatGPT | 0 | None identified | None | Not applicable | No clinically meaningful hallucination identified after audit. | Not applicable. | No hallucination identified. |
| 77 | Management | DeepSeek | 0 | None identified | None | Not applicable | No clinically meaningful hallucination identified after audit. | Not applicable. | No hallucination identified. |
| 77 | Management | Perplexity | 0 | None identified | None | Not applicable | No clinically meaningful hallucination identified after audit. | Not applicable. | No hallucination identified. |
| 77 | Management | Copilot | 0 | None identified | None | Not applicable | No clinically meaningful hallucination identified after audit. | Not applicable. | No hallucination identified. |
| 78 | Management | ChatGPT | 0 | None identified | None | Not applicable | No clinically meaningful hallucination identified after audit. | Not applicable. | No hallucination identified. |
| 78 | Management | DeepSeek | 0 | None identified | None | Not applicable | No clinically meaningful hallucination identified after audit. | Not applicable. | No hallucination identified. |
| 78 | Management | Perplexity | 0 | None identified | None | Not applicable | No clinically meaningful hallucination identified after audit. | Not applicable. | No hallucination identified. |
| 78 | Management | Copilot | 0 | None identified | None | Not applicable | No clinically meaningful hallucination identified after audit. | Not applicable. | No hallucination identified. |
| 79 | Management | ChatGPT | 0 | None identified | None | Not applicable | No clinically meaningful hallucination identified after audit. | Not applicable. | No hallucination identified. |
| 79 | Management | DeepSeek | 0 | None identified | None | Not applicable | No clinically meaningful hallucination identified after audit. | Not applicable. | No hallucination identified. |
| 79 | Management | Perplexity | 0 | None identified | None | Not applicable | No clinically meaningful hallucination identified after audit. | Not applicable. | No hallucination identified. |
| 79 | Management | Copilot | 0 | None identified | None | Not applicable | No clinically meaningful hallucination identified after audit. | Not applicable. | No hallucination identified. |
| 80 | Management | ChatGPT | 0 | None identified | None | Not applicable | No clinically meaningful hallucination identified after audit. | Not applicable. | No hallucination identified. |
| 80 | Management | DeepSeek | 0 | None identified | None | Not applicable | No clinically meaningful hallucination identified after audit. | Not applicable. | No hallucination identified. |
| 80 | Management | Perplexity | 0 | None identified | None | Not applicable | No clinically meaningful hallucination identified after audit. | Not applicable. | No hallucination identified. |
| 80 | Management | Copilot | 0 | None identified | None | Not applicable | No clinically meaningful hallucination identified after audit. | Not applicable. | No hallucination identified. |
| 81 | Management | ChatGPT | 1 | Unsupported supplement benefit | Moderate | Vitamin D, B vitamins, magnesium, and possibly CoQ10 may support HF management | May imply unproven supplement treatment benefit beyond correcting documented deficiency. | 2022 AHA/ACC/HFSA HF Guideline, guideline-directed medical therapy sections: vitamins, magnesium, CoQ10, and similar supplements are not guideline-directed HF treatments except where a documented deficiency or separate clinical indication exists; evidence is insufficient to present them as HF management therapies. | Hallucination identified, moderate potential severity. |
| 81 | Management | DeepSeek | 1 | Unsupported supplement benefit | Moderate | CoQ10 and vitamins may support HF management; Q-SYMBIO reduced symptoms/hospitalizations | Non-guideline supplements are presented as potentially beneficial HF interventions. | 2022 AHA/ACC/HFSA HF Guideline, guideline-directed medical therapy sections: vitamins, magnesium, CoQ10, and similar supplements are not guideline-directed HF treatments except where a documented deficiency or separate clinical indication exists; evidence is insufficient to present them as HF management therapies. | Hallucination identified, moderate potential severity. |
| 81 | Management | Perplexity | 1 | Unsupported vitamin benefit | Moderate | Vitamin D supplementation improves cardiac function and favorable remodeling | Vitamin D/supplements are not guideline-directed HF treatments except deficiency correction. | 2022 AHA/ACC/HFSA HF Guideline, guideline-directed medical therapy sections: vitamins, magnesium, CoQ10, and similar supplements are not guideline-directed HF treatments except where a documented deficiency or separate clinical indication exists; evidence is insufficient to present them as HF management therapies. | Hallucination identified, moderate potential severity. |
| 81 | Management | Copilot | 1 | Unsupported supplement benefit | Moderate | Several vitamins and supplements may benefit HF, reduce inflammation, and improve energy metabolism | Implies non-guideline supplements provide HF treatment benefit. | 2022 AHA/ACC/HFSA HF Guideline, guideline-directed medical therapy sections: vitamins, magnesium, CoQ10, and similar supplements are not guideline-directed HF treatments except where a documented deficiency or separate clinical indication exists; evidence is insufficient to present them as HF management therapies. | Hallucination identified, moderate potential severity. |
| 82 | Management | ChatGPT | 0 | None identified | None | Not applicable | No clinically meaningful hallucination identified after audit. | Not applicable. | No hallucination identified. |
| 82 | Management | DeepSeek | 0 | None identified | None | Not applicable | No clinically meaningful hallucination identified after audit. | Not applicable. | No hallucination identified. |
| 82 | Management | Perplexity | 1 | Unsupported statin mortality/HF benefit | Moderate | Statins may reduce death, cardiovascular events, and hospitalizations in HFrEF; benefits appear in ischemic and non-ischemic HF | Routine statin benefit for HF itself is not established; statins are indicated for ASCVD-related reasons. | 2022 AHA/ACC/HFSA HF Guideline and lipid/ASCVD prevention principles: statins are used for ASCVD-related indications and are not recommended as routine therapy to reduce mortality or HF hospitalization solely because a patient has HFrEF. | Hallucination identified, moderate potential severity. |
| 82 | Management | Copilot | 0 | None identified | None | Not applicable | No clinically meaningful hallucination identified after audit. | Not applicable. | No hallucination identified. |
| 83 | Management | ChatGPT | 1 | Overgeneralized iron supplementation benefit | Low | Iron supplements, particularly IV iron, are recommended for HF patients with confirmed iron deficiency | Directionally correct for selected iron-deficient symptomatic patients, but broad wording/“supplements” may overgeneralize. | 2022 AHA/ACC/HFSA HF Guideline, guideline-directed medical therapy sections: vitamins, magnesium, CoQ10, and similar supplements are not guideline-directed HF treatments except where a documented deficiency or separate clinical indication exists; evidence is insufficient to present them as HF management therapies. | Hallucination identified, low potential severity. |
| 83 | Management | DeepSeek | 0 | None identified | None | Not applicable | No clinically meaningful hallucination identified after audit. | Not applicable. | No hallucination identified. |
| 83 | Management | Perplexity | 1 | Overgeneralized iron benefit | Low | IV or oral iron supplementation improves symptoms and may reduce hospitalizations | Guideline-supported benefit is primarily IV iron for selected iron-deficient HF patients; oral iron benefit is not established. | 2022 AHA/ACC/HFSA HF Guideline, iron deficiency recommendations: intravenous iron may be considered for selected symptomatic HF patients with confirmed iron deficiency; oral iron and broad “iron supplements” should not be generalized as improving HF outcomes. | Hallucination identified, low potential severity. |
| 83 | Management | Copilot | 1 | Overgeneralized iron-deficiency prevalence/survival claim | Low | Iron deficiency affects up to 50–80% of HF patients and is linked to reduced survival | Broad prevalence/survival framing should specify selected IV iron indications. | 2022 AHA/ACC/HFSA HF Guideline, iron deficiency recommendations: intravenous iron may be considered for selected symptomatic HF patients with confirmed iron deficiency; oral iron and broad “iron supplements” should not be generalized as improving HF outcomes. | Hallucination identified, low potential severity. |
| 84 | Management | ChatGPT | 0 | None identified | None | Not applicable | No clinically meaningful hallucination identified after audit. | Not applicable. | No hallucination identified. |
| 84 | Management | DeepSeek | 0 | None identified | None | Not applicable | No clinically meaningful hallucination identified after audit. | Not applicable. | No hallucination identified. |
| 84 | Management | Perplexity | 1 | Overgeneralized anticoagulation rationale | Low | HF patients without AF may require anticoagulation if they have high stroke risk | Anticoagulation is not recommended for HF alone without a clear indication. | 2022 AHA/ACC/HFSA HF Guideline, anticoagulation recommendations: anticoagulation is not recommended for chronic HF alone without a specific indication such as atrial fibrillation, venous thromboembolism, mechanical valve, or other established thromboembolic risk indication. | Hallucination identified, low potential severity. |
| 84 | Management | Copilot | 0 | None identified | None | Not applicable | No clinically meaningful hallucination identified after audit. | Not applicable. | No hallucination identified. |
| 85 | Management | ChatGPT | 0 | None identified | None | Not applicable | No clinically meaningful hallucination identified after audit. | Not applicable. | No hallucination identified. |
| 85 | Management | DeepSeek | 0 | None identified | None | Not applicable | No clinically meaningful hallucination identified after audit. | Not applicable. | No hallucination identified. |
| 85 | Management | Perplexity | 0 | None identified | None | Not applicable | No clinically meaningful hallucination identified after audit. | Not applicable. | No hallucination identified. |
| 85 | Management | Copilot | 0 | None identified | None | Not applicable | No clinically meaningful hallucination identified after audit. | Not applicable. | No hallucination identified. |
| 86 | Management | ChatGPT | 0 | None identified | None | Not applicable | No clinically meaningful hallucination identified after audit. | Not applicable. | No hallucination identified. |
| 86 | Management | DeepSeek | 0 | None identified | None | Not applicable | No clinically meaningful hallucination identified after audit. | Not applicable. | No hallucination identified. |
| 86 | Management | Perplexity | 0 | None identified | None | Not applicable | No clinically meaningful hallucination identified after audit. | Not applicable. | No hallucination identified. |
| 86 | Management | Copilot | 0 | None identified | None | Not applicable | No clinically meaningful hallucination identified after audit. | Not applicable. | No hallucination identified. |
| 87 | Management | ChatGPT | 0 | None identified | None | Not applicable | No clinically meaningful hallucination identified after audit. | Not applicable. | No hallucination identified. |
| 87 | Management | DeepSeek | 0 | None identified | None | Not applicable | No clinically meaningful hallucination identified after audit. | Not applicable. | No hallucination identified. |
| 87 | Management | Perplexity | 0 | None identified | None | Not applicable | No clinically meaningful hallucination identified after audit. | Not applicable. | No hallucination identified. |
| 87 | Management | Copilot | 0 | None identified | None | Not applicable | No clinically meaningful hallucination identified after audit. | Not applicable. | No hallucination identified. |
| 88 | Management | ChatGPT | 0 | None identified | None | Not applicable | No clinically meaningful hallucination identified after audit. | Not applicable. | No hallucination identified. |
| 88 | Management | DeepSeek | 0 | None identified | None | Not applicable | No clinically meaningful hallucination identified after audit. | Not applicable. | No hallucination identified. |
| 88 | Management | Perplexity | 0 | None identified | None | Not applicable | No clinically meaningful hallucination identified after audit. | Not applicable. | No hallucination identified. |
| 88 | Management | Copilot | 0 | None identified | None | Not applicable | No clinically meaningful hallucination identified after audit. | Not applicable. | No hallucination identified. |
| 89 | Management | ChatGPT | 1 | Non-HF-specific lipid target | Low | Aim for LDL cholesterol below 70 mg/dL if HF with CAD | LDL targets are ASCVD-risk based, not HF-specific. | 2022 AHA/ACC/HFSA HF Guideline and ASCVD lipid-management guidance: LDL targets are determined by ASCVD risk and comorbid indications, not by HF status itself; HF-specific LDL targets are not established in the HF guideline. | Hallucination identified, low potential severity. |
| 89 | Management | DeepSeek | 1 | Non-HF-specific lipid target | Low | High-risk HF patients with CAD/diabetes/CKD should aim for LDL <70 mg/dL | LDL goals are ASCVD-risk based rather than HF-specific. | 2022 AHA/ACC/HFSA HF Guideline and ASCVD lipid-management guidance: LDL targets are determined by ASCVD risk and comorbid indications, not by HF status itself; HF-specific LDL targets are not established in the HF guideline. | Hallucination identified, low potential severity. |
| 89 | Management | Perplexity | 1 | Non-HF-specific cholesterol target | Low | LDL <70 mg/dL for HF patients with ASCVD or very high risk | This is ASCVD-risk based, not HF-specific. | 2022 AHA/ACC/HFSA HF Guideline and ASCVD lipid-management guidance: LDL targets are determined by ASCVD risk and comorbid indications, not by HF status itself; HF-specific LDL targets are not established in the HF guideline. | Hallucination identified, low potential severity. |
| 89 | Management | Copilot | 1 | Non-HF-specific lipid target | Low | Recommended cholesterol levels in HF with ASCVD | Lipid targets are ASCVD-risk based rather than HF-specific. | 2022 AHA/ACC/HFSA HF Guideline and ASCVD lipid-management guidance: LDL targets are determined by ASCVD risk and comorbid indications, not by HF status itself; HF-specific LDL targets are not established in the HF guideline. | Hallucination identified, low potential severity. |
| 90 | Management | ChatGPT | 0 | None identified | None | Not applicable | No clinically meaningful hallucination identified after audit. | Not applicable. | No hallucination identified. |
| 90 | Management | DeepSeek | 0 | None identified | None | Not applicable | No clinically meaningful hallucination identified after audit. | Not applicable. | No hallucination identified. |
| 90 | Management | Perplexity | 0 | None identified | None | Not applicable | No clinically meaningful hallucination identified after audit. | Not applicable. | No hallucination identified. |
| 90 | Management | Copilot | 0 | None identified | None | Not applicable | No clinically meaningful hallucination identified after audit. | Not applicable. | No hallucination identified. |
| 91 | Management | ChatGPT | 1 | Overgeneralized testing indication | Low | Stress tests are useful before starting or adjusting certain therapies | Stress testing is selective and not routine for HF therapy adjustment. | 2022 AHA/ACC/HFSA HF Guideline, diagnostic evaluation for ischemic heart disease: stress testing is selective and based on suspected ischemia, viability assessment, or clinical decision needs; it is not routinely required for all HF patients or before ordinary HF therapy adjustment. | Hallucination identified, low potential severity. |
| 91 | Management | DeepSeek | 0 | None identified | None | Not applicable | No clinically meaningful hallucination identified after audit. | Not applicable. | No hallucination identified. |
| 91 | Management | Perplexity | 1 | Overgeneralized stress-test recommendation | Low | Stress testing is recommended for HF to evaluate exercise capacity and ischemia | Stress testing is selective, not routine for all HF patients. | 2022 AHA/ACC/HFSA HF Guideline, diagnostic evaluation for ischemic heart disease: stress testing is selective and based on suspected ischemia, viability assessment, or clinical decision needs; it is not routinely required for all HF patients or before ordinary HF therapy adjustment. | Hallucination identified, low potential severity. |
| 91 | Management | Copilot | 1 | Overgeneralized stress testing | Low | A stress test is often recommended for people with HF | Stress testing is selective rather than broadly recommended. | 2022 AHA/ACC/HFSA HF Guideline, diagnostic evaluation for ischemic heart disease: stress testing is selective and based on suspected ischemia, viability assessment, or clinical decision needs; it is not routinely required for all HF patients or before ordinary HF therapy adjustment. | Hallucination identified, low potential severity. |
| 92 | Other | ChatGPT | 0 | None identified | None | Not applicable | No clinically meaningful hallucination identified after audit. | Not applicable. | No hallucination identified. |
| 92 | Other | DeepSeek | 0 | None identified | None | Not applicable | No clinically meaningful hallucination identified after audit. | Not applicable. | No hallucination identified. |
| 92 | Other | Perplexity | 0 | None identified | None | Not applicable | No clinically meaningful hallucination identified after audit. | Not applicable. | No hallucination identified. |
| 92 | Other | Copilot | 0 | None identified | None | Not applicable | No clinically meaningful hallucination identified after audit. | Not applicable. | No hallucination identified. |
| 93 | Other | ChatGPT | 0 | None identified | None | Not applicable | No clinically meaningful hallucination identified after audit. | Not applicable. | No hallucination identified. |
| 93 | Other | DeepSeek | 0 | None identified | None | Not applicable | No clinically meaningful hallucination identified after audit. | Not applicable. | No hallucination identified. |
| 93 | Other | Perplexity | 0 | None identified | None | Not applicable | No clinically meaningful hallucination identified after audit. | Not applicable. | No hallucination identified. |
| 93 | Other | Copilot | 0 | None identified | None | Not applicable | No clinically meaningful hallucination identified after audit. | Not applicable. | No hallucination identified. |
| 94 | Other | ChatGPT | 0 | None identified | None | Not applicable | No clinically meaningful hallucination identified after audit. | Not applicable. | No hallucination identified. |
| 94 | Other | DeepSeek | 0 | None identified | None | Not applicable | No clinically meaningful hallucination identified after audit. | Not applicable. | No hallucination identified. |
| 94 | Other | Perplexity | 0 | None identified | None | Not applicable | No clinically meaningful hallucination identified after audit. | Not applicable. | No hallucination identified. |
| 94 | Other | Copilot | 0 | None identified | None | Not applicable | No clinically meaningful hallucination identified after audit. | Not applicable. | No hallucination identified. |
| 95 | Other | ChatGPT | 0 | None identified | None | Not applicable | No clinically meaningful hallucination identified after audit. | Not applicable. | No hallucination identified. |
| 95 | Other | DeepSeek | 0 | None identified | None | Not applicable | No clinically meaningful hallucination identified after audit. | Not applicable. | No hallucination identified. |
| 95 | Other | Perplexity | 0 | None identified | None | Not applicable | No clinically meaningful hallucination identified after audit. | Not applicable. | No hallucination identified. |
| 95 | Other | Copilot | 0 | None identified | None | Not applicable | No clinically meaningful hallucination identified after audit. | Not applicable. | No hallucination identified. |
| 96 | Other | ChatGPT | 0 | None identified | None | Not applicable | No clinically meaningful hallucination identified after audit. | Not applicable. | No hallucination identified. |
| 96 | Other | DeepSeek | 0 | None identified | None | Not applicable | No clinically meaningful hallucination identified after audit. | Not applicable. | No hallucination identified. |
| 96 | Other | Perplexity | 0 | None identified | None | Not applicable | No clinically meaningful hallucination identified after audit. | Not applicable. | No hallucination identified. |
| 96 | Other | Copilot | 0 | None identified | None | Not applicable | No clinically meaningful hallucination identified after audit. | Not applicable. | No hallucination identified. |
| 97 | Other | ChatGPT | 0 | None identified | None | Not applicable | No clinically meaningful hallucination identified after audit. | Not applicable. | No hallucination identified. |
| 97 | Other | DeepSeek | 0 | None identified | None | Not applicable | No clinically meaningful hallucination identified after audit. | Not applicable. | No hallucination identified. |
| 97 | Other | Perplexity | 0 | None identified | None | Not applicable | No clinically meaningful hallucination identified after audit. | Not applicable. | No hallucination identified. |
| 97 | Other | Copilot | 0 | None identified | None | Not applicable | No clinically meaningful hallucination identified after audit. | Not applicable. | No hallucination identified. |
| 98 | Other | ChatGPT | 0 | None identified | None | Not applicable | No clinically meaningful hallucination identified after audit. | Not applicable. | No hallucination identified. |
| 98 | Other | DeepSeek | 0 | None identified | None | Not applicable | No clinically meaningful hallucination identified after audit. | Not applicable. | No hallucination identified. |
| 98 | Other | Perplexity | 0 | None identified | None | Not applicable | No clinically meaningful hallucination identified after audit. | Not applicable. | No hallucination identified. |
| 98 | Other | Copilot | 0 | None identified | None | Not applicable | No clinically meaningful hallucination identified after audit. | Not applicable. | No hallucination identified. |
| 99 | Other | ChatGPT | 0 | None identified | None | Not applicable | No clinically meaningful hallucination identified after audit. | Not applicable. | No hallucination identified. |
| 99 | Other | DeepSeek | 0 | None identified | None | Not applicable | No clinically meaningful hallucination identified after audit. | Not applicable. | No hallucination identified. |
| 99 | Other | Perplexity | 0 | None identified | None | Not applicable | No clinically meaningful hallucination identified after audit. | Not applicable. | No hallucination identified. |
| 99 | Other | Copilot | 0 | None identified | None | Not applicable | No clinically meaningful hallucination identified after audit. | Not applicable. | No hallucination identified. |
| 100 | Other | ChatGPT | 0 | None identified | None | Not applicable | No clinically meaningful hallucination identified after audit. | Not applicable. | No hallucination identified. |
| 100 | Other | DeepSeek | 0 | None identified | None | Not applicable | No clinically meaningful hallucination identified after audit. | Not applicable. | No hallucination identified. |
| 100 | Other | Perplexity | 1 | Over-specific cause-of-death ranking | Low | Progressive pump failure is the most common cause of death and accounts for about 24% | Cause of death varies by EF, age, disease stage, and therapy; a single percentage may mislead. | 2022 AHA/ACC/HFSA HF Guideline and HF epidemiology evidence: cause of death varies by ejection fraction, age, comorbidity burden, disease stage, and therapy; a single ranking or fixed percentage for progressive pump failure is not generalizable. | Hallucination identified, low potential severity. |
| 100 | Other | Copilot | 0 | None identified | None | Not applicable | No clinically meaningful hallucination identified after audit. | Not applicable. | No hallucination identified. |
| 101 | Other | ChatGPT | 0 | None identified | None | Not applicable | No clinically meaningful hallucination identified after audit. | Not applicable. | No hallucination identified. |
| 101 | Other | DeepSeek | 0 | None identified | None | Not applicable | No clinically meaningful hallucination identified after audit. | Not applicable. | No hallucination identified. |
| 101 | Other | Perplexity | 0 | None identified | None | Not applicable | No clinically meaningful hallucination identified after audit. | Not applicable. | No hallucination identified. |
| 101 | Other | Copilot | 0 | None identified | None | Not applicable | No clinically meaningful hallucination identified after audit. | Not applicable. | No hallucination identified. |
| 102 | Other | ChatGPT | 0 | None identified | None | Not applicable | No clinically meaningful hallucination identified after audit. | Not applicable. | No hallucination identified. |
| 102 | Other | DeepSeek | 0 | None identified | None | Not applicable | No clinically meaningful hallucination identified after audit. | Not applicable. | No hallucination identified. |
| 102 | Other | Perplexity | 1 | Overstated ablation outcome benefit | Low | AF ablation may reduce mortality and hospitalizations in HF | Benefit applies to selected AF/HFrEF patients and should not be generalized to HF patients. | 2022 AHA/ACC/HFSA HF Guideline and AF/HF evidence: catheter ablation may benefit selected patients with AF and HF, particularly selected HFrEF populations, but mortality and hospitalization benefits should not be generalized to all HF patients. | Hallucination identified, low potential severity. |
| 102 | Other | Copilot | 0 | None identified | None | Not applicable | No clinically meaningful hallucination identified after audit. | Not applicable. | No hallucination identified. |
| 103 | Other | ChatGPT | 0 | None identified | None | Not applicable | No clinically meaningful hallucination identified after audit. | Not applicable. | No hallucination identified. |
| 103 | Other | DeepSeek | 0 | None identified | None | Not applicable | No clinically meaningful hallucination identified after audit. | Not applicable. | No hallucination identified. |
| 103 | Other | Perplexity | 0 | None identified | None | Not applicable | No clinically meaningful hallucination identified after audit. | Not applicable. | No hallucination identified. |
| 103 | Other | Copilot | 0 | None identified | None | Not applicable | No clinically meaningful hallucination identified after audit. | Not applicable. | No hallucination identified. |
| 104 | Other | ChatGPT | 0 | None identified | None | Not applicable | No clinically meaningful hallucination identified after audit. | Not applicable. | No hallucination identified. |
| 104 | Other | DeepSeek | 0 | None identified | None | Not applicable | No clinically meaningful hallucination identified after audit. | Not applicable. | No hallucination identified. |
| 104 | Other | Perplexity | 0 | None identified | None | Not applicable | No clinically meaningful hallucination identified after audit. | Not applicable. | No hallucination identified. |
| 104 | Other | Copilot | 0 | None identified | None | Not applicable | No clinically meaningful hallucination identified after audit. | Not applicable. | No hallucination identified. |
| 105 | Other | ChatGPT | 0 | None identified | None | Not applicable | No clinically meaningful hallucination identified after audit. | Not applicable. | No hallucination identified. |
| 105 | Other | DeepSeek | 0 | None identified | None | Not applicable | No clinically meaningful hallucination identified after audit. | Not applicable. | No hallucination identified. |
| 105 | Other | Perplexity | 0 | None identified | None | Not applicable | No clinically meaningful hallucination identified after audit. | Not applicable. | No hallucination identified. |
| 105 | Other | Copilot | 1 | Imprecise CRT threshold | Low | CRT recommended for QRS duration ≥130 ms with HFrEF and conduction delay | CRT indications depend on LVEF, rhythm, LBBB morphology, QRS duration categories, and symptoms. | 2022 AHA/ACC/HFSA HF Guideline, cardiac resynchronization therapy recommendations: CRT eligibility depends on LVEF, rhythm, LBBB morphology, QRS duration, NYHA class, and GDMT status; QRS duration alone is an incomplete indication. | Hallucination identified, low potential severity. |
| 106 | Other | ChatGPT | 0 | None identified | None | Not applicable | No clinically meaningful hallucination identified after audit. | Not applicable. | No hallucination identified. |
| 106 | Other | DeepSeek | 0 | None identified | None | Not applicable | No clinically meaningful hallucination identified after audit. | Not applicable. | No hallucination identified. |
| 106 | Other | Perplexity | 0 | None identified | None | Not applicable | No clinically meaningful hallucination identified after audit. | Not applicable. | No hallucination identified. |
| 106 | Other | Copilot | 0 | None identified | None | Not applicable | No clinically meaningful hallucination identified after audit. | Not applicable. | No hallucination identified. |
| 107 | Other | ChatGPT | 0 | None identified | None | Not applicable | No clinically meaningful hallucination identified after audit. | Not applicable. | No hallucination identified. |
| 107 | Other | DeepSeek | 0 | None identified | None | Not applicable | No clinically meaningful hallucination identified after audit. | Not applicable. | No hallucination identified. |
| 107 | Other | Perplexity | 0 | None identified | None | Not applicable | No clinically meaningful hallucination identified after audit. | Not applicable. | No hallucination identified. |
| 107 | Other | Copilot | 0 | None identified | None | Not applicable | No clinically meaningful hallucination identified after audit. | Not applicable. | No hallucination identified. |
| 108 | Other | ChatGPT | 1 | Overgeneralized device benefit | Low | CardioMEMS reduces HF admissions by about 44–57% and improves quality of life | Benefit depends on selected populations/trials; phrasing may overgeneralize. | 2022 AHA/ACC/HFSA HF Guideline, wireless pulmonary artery pressure monitoring recommendations: CardioMEMS/pulmonary artery pressure monitoring is considered for selected patients, and its benefits depend on trial population and eligibility criteria; broad claims of universal HFrEF/HFpEF benefit or cost-effectiveness are overgeneralized. | Hallucination identified, low potential severity. |
| 108 | Other | DeepSeek | 1 | Over-specific/partly inaccurate CardioMEMS eligibility | Low | All 3 criteria must be met: NYHA III, HF hospitalization, and elevated NT-proBNP/BNP | Eligibility criteria are presented too rigidly for selected-use pulmonary artery pressure monitoring. | 2022 AHA/ACC/HFSA HF Guideline, wireless pulmonary artery pressure monitoring recommendations: CardioMEMS/pulmonary artery pressure monitoring is considered for selected patients, and its benefits depend on trial population and eligibility criteria; broad claims of universal HFrEF/HFpEF benefit or cost-effectiveness are overgeneralized. | Hallucination identified, low potential severity. |
| 108 | Other | Perplexity | 1 | Overgeneralized device benefit/cost-effectiveness | Low | CardioMEMS reduces admissions by 44–57%, improves quality of life, and is cost-effective | Benefits/cost-effectiveness are population- and trial-dependent. | 2022 AHA/ACC/HFSA HF Guideline, wireless pulmonary artery pressure monitoring recommendations: CardioMEMS/pulmonary artery pressure monitoring is considered for selected patients, and its benefits depend on trial population and eligibility criteria; broad claims of universal HFrEF/HFpEF benefit or cost-effectiveness are overgeneralized. | Hallucination identified, low potential severity. |
| 108 | Other | Copilot | 1 | Overgeneralized CardioMEMS benefit | Low | Works for both HFrEF and HFpEF and is proven effective; reduces hospitalizations 37–50% | CardioMEMS benefit is for selected patients; wording overgeneralizes broad HFrEF/HFpEF effectiveness. | 2022 AHA/ACC/HFSA HF Guideline, wireless pulmonary artery pressure monitoring recommendations: CardioMEMS/pulmonary artery pressure monitoring is considered for selected patients, and its benefits depend on trial population and eligibility criteria; broad claims of universal HFrEF/HFpEF benefit or cost-effectiveness are overgeneralized. | Hallucination identified, low potential severity. |

*Note. Hallucination was assessed at the response level. A response was classified as containing a hallucination if it included at least one clinically meaningful statement inconsistent with the 2022 AHA/ACC/HFSA guideline, contradicted established clinical evidence, was unsupported by applicable evidence, was overgeneralized beyond the appropriate patient population, or could mislead patient-facing HF education. Among the 40 responses classified as hallucinated, 33 were rated as low potential severity and 7 were rated as moderate potential severity; no high-severity hallucinations were identified. Detailed flagged statement excerpts, audit rationales, reference standards, and final adjudicated decisions are provided to support response-level reproducibility.*

Supplementary Table S7. Paired binary analyses of response-level hallucination outcomes.

*The same 108 questions were answered by all four chatbot systems. Therefore, paired binary methods were used to account for the repeated question-level structure.*

S7A. Overall paired binary tests.

| **Test** | **Test statistic** | **df** | **P value** | **Interpretation** |
| --- | --- | --- | --- | --- |
| Cochran's Q test | 15.273 | 3 | 0.002 | Paired binary comparison across the four chatbot systems. |
| GEE logistic regression, exchangeable correlation | 12.384 | 3 | 0.006 | Accounts for question-level clustering. |

S7B. GEE logistic regression coefficients.

| **Term** | **OR (95% CI)** | **P value** |
| --- | --- | --- |
| ChatGPT vs DeepSeek | 1.360 (0.745-2.484) | 0.317 |
| Copilot vs DeepSeek | 1.735 (0.895-3.360) | 0.103 |
| Perplexity vs DeepSeek | 2.957 (1.510-5.788) | 0.002 |

## S7C. Exact pairwise McNemar tests.

| **Comparison** | **n00 both no** | **n01 model1 no / model2 yes** | **n10 model1 yes / model2 no** | **n11 both yes** | **Exact McNemar P** | **Bonferroni-adjusted P** | **Holm-adjusted P** | **Significant after Bonferroni** |
| --- | --- | --- | --- | --- | --- | --- | --- | --- |
| ChatGPT vs DeepSeek | 99 | 1 | 3 | 5 | 0.625 | 1.000 | 1.000 | No |
| ChatGPT vs Perplexity | 92 | 8 | 0 | 8 | 0.008 | 0.047 | 0.039 | Yes |
| ChatGPT vs Copilot | 97 | 3 | 1 | 7 | 0.625 | 1.000 | 1.000 | No |
| DeepSeek vs Perplexity | 92 | 10 | 0 | 6 | 0.002 | 0.012 | 0.012 | Yes |
| DeepSeek vs Copilot | 97 | 5 | 1 | 5 | 0.219 | 1.000 | 0.656 | No |
| Perplexity vs Copilot | 89 | 3 | 9 | 7 | 0.146 | 0.876 | 0.584 | No |

*Note. In pairwise McNemar tests, n01 indicates responses classified as non-hallucinated for model 1 but hallucinated for model 2; n10 indicates responses classified as hallucinated for model 1 but non-hallucinated for model 2. Bonferroni-adjusted P values were calculated by multiplying each exact McNemar P value by six pairwise comparisons and truncating values above 1.000 to 1.000. DeepSeek was used as the reference category in the GEE logistic regression model; OR > 1 indicates higher odds of response-level hallucination compared with DeepSeek.*

Supplementary Table S8. Inter-rater reliability and internal consistency of human-rated outcomes.

This table summarizes pre-consensus agreement for the human-rated quality instruments and the response-level hallucination audit. Disagreements were resolved by discussion and, when needed, adjudicated by a senior cardiologist.

| **Outcome** | **Raters/items** | **Statistic** | **Estimate** | **Assessment stage** | **Interpretation** |
| --- | --- | --- | --- | --- | --- |
| mDISCERN score | Three raters | ICC(2,k), two-way random-effects model, absolute agreement | 0.870 | Before consensus | Good / almost perfect agreement |
| EQIP percentage score | Three raters | ICC(2,k), two-way random-effects model, absolute agreement | 0.830 | Before consensus | Good / almost perfect agreement |
| Response-level hallucination classification | Two cardiovascular-trained reviewers | Cohen's κ | 0.840 | Before adjudication | Substantial to excellent agreement |
| mDISCERN internal consistency | 12-item mDISCERN instrument | Cronbach's α | 0.870 | Scale-level internal consistency | Good internal consistency |

Note. ICC(2,k) indicates a two-way random-effects intraclass correlation coefficient for absolute agreement based on the average rating of k raters. κ, kappa. Agreement statistics were calculated before consensus discussion or adjudication.

Supplementary Figure S1. Secondary readability metrics across chatbot systems.

*Bar chart comparisons of secondary readability metrics across the four LLM-based chatbot systems, stratified by question category. Error bars represent 95% confidence intervals. Abbreviations: ARI, Automated Readability Index; FRES, Flesch Reading Ease Score; GFOG, Gunning Fog Index; FKGL, Flesch-Kincaid Grade Level; CLI, Coleman-Liau Index.*

Supplementary Figure S1A. Calculator 1 secondary readability metrics.

Supplementary Figure S1B. Calculator 2 secondary readability metrics.

# Appendix A. Full list of 108 standardized heart failure questions.

*Questions were categorized into three domains: basic knowledge (n = 50), management (n = 41), and other HF-related issues (n = 17).*

| **Question ID** | **Category** | **Question** |
| --- | --- | --- |
| 1 | Basic knowledge | Can heart failure affect other organs in the body? |
| 2 | Basic knowledge | How does heart failure affect the lungs? |
| 3 | Basic knowledge | How does heart failure affect the kidneys? |
| 4 | Basic knowledge | What is cardiomyopathy and how does it relate to heart failure? |
| 5 | Basic knowledge | Can you be born with heart failure? |
| 6 | Basic knowledge | Can viruses cause heart failure? |
| 7 | Basic knowledge | What are common causes of heart failure? |
| 8 | Basic knowledge | Is heart failure genetically related? |
| 9 | Basic knowledge | Does atrial fibrillation cause heart failure? |
| 10 | Basic knowledge | Can heart failure be caused by an autoimmune disease? |
| 11 | Basic knowledge | Can you develop heart failure from pregnancy? |
| 12 | Basic knowledge | What are the different types of heart failure? |
| 13 | Basic knowledge | Can a heart attack cause heart failure? |
| 14 | Basic knowledge | Can diabetes cause heart failure? |
| 15 | Basic knowledge | Can old age cause heart failure? |
| 16 | Basic knowledge | Can COVID-19 cause heart failure? |
| 17 | Basic knowledge | Can lung disease cause heart failure? |
| 18 | Basic knowledge | Is heart failure more common in men or women? |
| 19 | Basic knowledge | Can kidney disease cause heart failure? |
| 20 | Basic knowledge | Does anemia make heart failure worse? |
| 21 | Basic knowledge | Can stress cause heart failure? |
| 22 | Basic knowledge | How does amyloidosis cause heart failure? |
| 23 | Basic knowledge | Why does pulmonary edema occur in heart failure? |
| 24 | Basic knowledge | I have heart failure. What should I do if I have chest pain? |
| 25 | Basic knowledge | How can appetite be improved in heart failure? |
| 26 | Basic knowledge | What can an echocardiogram tell me about my heart failure? |
| 27 | Basic knowledge | How is heart failure diagnosed? |
| 28 | Basic knowledge | What is left sided heart failure? |
| 29 | Basic knowledge | What is right sided heart failure? |
| 30 | Basic knowledge | What does diastolic heart failure mean? |
| 31 | Basic knowledge | What does systolic heart failure mean? |
| 32 | Basic knowledge | What is combined systolic and diastolic heart failure? |
| 33 | Basic knowledge | What does a high BNP mean? |
| 34 | Basic knowledge | What does biventricular heart failure mean? |
| 35 | Basic knowledge | How common is heart failure? |
| 36 | Basic knowledge | What are the AHA/ACC stages of heart failure? |
| 37 | Basic knowledge | What are the NYHA classes of heart failure? |
| 38 | Basic knowledge | What does a high NT-proBNP mean? |
| 39 | Basic knowledge | What does a high ST-2 mean? |
| 40 | Basic knowledge | What does a high LVEDP mean? |
| 41 | Basic knowledge | What ejection fraction number is considered heart failure? |
| 42 | Basic knowledge | What symptoms are related to heart failure? |
| 43 | Basic knowledge | What heart failure symptoms should I watch for and when should I go to the hospital? |
| 44 | Basic knowledge | I have heart failure and am taking diuretics. How can I stop needing to use the bathroom so much in the middle of the night? |
| 45 | Basic knowledge | I have heart failure. How can I improve my breathing at night when I am sleeping? |
| 46 | Basic knowledge | What do I do if I get shocked by my defibrillator that I have for heart failure? |
| 47 | Basic knowledge | What are common symptoms of heart failure? |
| 48 | Basic knowledge | I have heart failure. What should I do if my weight increases significantly in a short amount of time? |
| 49 | Basic knowledge | Why does heart failure cause more frequent coughing? |
| 50 | Basic knowledge | How can fatigue be improved in someone with heart failure? |
| 51 | Management | Will smoking cigarettes make heart failure worse? |
| 52 | Management | Will drinking alcohol make heart failure worse? |
| 53 | Management | What strategies have other patients with heart failure found useful for motivating themselves to eat healthier, exercise, stop smoking and make other lifestyle changes? |
| 54 | Management | If my ejection fraction improves do I still need take my heart failure medications? |
| 55 | Management | When would I need a Zoll life vest for heart failure? |
| 56 | Management | What is guideline directed medical therapy for heart failure? |
| 57 | Management | What is diuretic resistance in heart failure? |
| 58 | Management | Is it safe to take multiple diuretics for heart failure? |
| 59 | Management | What is the recommended treatment for heart failure with atrial fibrillation? |
| 60 | Management | How does a mineralocorticoid antagonist treat heart failure? |
| 61 | Management | When would I need an AICD for heart failure? |
| 62 | Management | What risk factors should I control to prevent heart failure? |
| 63 | Management | What are the benefits of a cardiac rehabilitation program for heart failure? |
| 64 | Management | How much salt intake is recommended in a day for someone with heart failure? |
| 65 | Management | How much water intake is recommended in a day for someone with heart failure? |
| 66 | Management | How much exercise each week is recommended for someone with heart failure? |
| 67 | Management | What diet is recommended for heart failure? |
| 68 | Management | Why is blood pressure important to control in heart failure? |
| 69 | Management | Does losing weight help heart failure? |
| 70 | Management | What blood glucose is recommended for someone with heart failure? |
| 71 | Management | Does home oxygen help heart failure? |
| 72 | Management | How often should I check my blood pressure for heart failure? |
| 73 | Management | Are there activities or exercises that are not safe for someone with heart failure to do? |
| 74 | Management | Are there limits on what I can do at work if I have heart failure? |
| 75 | Management | Is it safe to be sexually active in heart failure? |
| 76 | Management | Are compression socks recommended for heart failure? |
| 77 | Management | How does a beta blocker treat heart failure? |
| 78 | Management | How does Jardiance treat heart failure? |
| 79 | Management | What should I do if I miss a dose of my heart failure medication? |
| 80 | Management | What over-the-counter medicines are not recommended in someone with heart failure? |
| 81 | Management | What vitamins are beneficial for heart failure? |
| 82 | Management | Are statins recommended for heart failure? |
| 83 | Management | Are iron supplements recommended for heart failure? |
| 84 | Management | Is anticoagulation recommended for heart failure? |
| 85 | Management | Is it safe to use sildenafil (Viagra), vardenafil (Levitra), or tadalafil (Cialis) in heart failure? |
| 86 | Management | How often do you need to obtain an electrocardiogram for heart failure? |
| 87 | Management | How often do you need an echocardiogram for heart failure? |
| 88 | Management | How often should I check my weight for heart failure? |
| 89 | Management | What cholesterol level is recommended for heart failure? |
| 90 | Management | Are disability benefits available for heart failure? |
| 91 | Management | Is a stress test recommended for heart failure? |
| 92 | Other | Am I at higher risk of having a heart attack because of my heart failure? |
| 93 | Other | Is there a cure for heart failure? |
| 94 | Other | What is the life expectancy for someone newly diagnosed with heart failure? |
| 95 | Other | How can the progression of heart failure be slowed down? |
| 96 | Other | Am I at higher risk of stroke due to heart failure? |
| 97 | Other | After being hospitalized for heart failure, how can I prevent needing to go back? |
| 98 | Other | How can I support my family member with heart failure? |
| 99 | Other | How will my heart failure diagnosis impact my family? |
| 100 | Other | What is the most common cause of death in heart failure? |
| 101 | Other | Is a cardiac catheterization recommended for heart failure? |
| 102 | Other | Is an ablation recommended for heart failure? |
| 103 | Other | When would someone need a heart transplant for heart failure? |
| 104 | Other | What is an LVAD? |
| 105 | Other | When is a pacemaker recommended for heart failure? |
| 106 | Other | Am I at higher risk for infection from heart failure? |
| 107 | Other | How can I improve my depression due to heart failure? |
| 108 | Other | What are the benefits of a CardioMEMS procedure? |

# Appendix B. Scoring templates and definitions.

B1. Ensuring Quality Information for Patients (EQIP; 20-item tool)

Each EQIP item was scored as Yes, Partially, No, or Not applicable. In this study, Yes, Partially, and No were assigned values of 1, 0.5, and 0, respectively. Not applicable items were removed from the denominator when calculating the final percentage score. EQIP scores were analyzed as percentages on a 0-100 scale. The 20 items used for scoring are listed below for reproducibility.

| **Domain** | **Item** | **Question** |
| --- | --- | --- |
| Content/structure | 1 | Does the document start by telling you what it will cover and then cover what it says? |
| Language | 2 | Does it use everyday language, explaining unusual or medical words, abbreviations, or jargon? |
| Language | 3 | Does it use short sentences of less than 15 words on average? |
| Tone/patient orientation | 4 | Does it personally address the reader? |
| Tone/patient orientation | 5 | Is the tone respectful? |
| Design/layout | 6 | Is the design and layout of the document satisfactory? |
| Design/layout | 7 | Does the document contain easy-to-understand illustrations, diagrams, or photos that are relevant to the subjects it covers? |
| Structure | 8 | Is the information presented in a logical order? |
| Actionability | 9 | Does the document have a named space for the reader to make notes? |
| Actionability/contact | 10 | Does the document contain contact details for the healthcare services where the reader can receive care or treatment for problems discussed in the document? |
| Identification | 11 | Does the document contain the date it was produced? |
| Identification | 12 | Does the document contain the name of the person or department that produced it? |
| Patient involvement | 13 | Does the document say whether patients and/or their families were involved or consulted in its production? |
| Medication/product naming | 14 | Does it use generic names for all medications or products instead of, or in addition to, brand names and designate brand names as such? |
| Content | 15 | Does the document address quality-of-life issues, such as school attendance, work, reduced mobility, or daily activities, when relevant? |
| Further information | 16 | Does the document contain details of other sources of information for the reader, such as support organizations or websites? |
| Purpose | 17 | Is the purpose described? |
| Benefits | 18 | Are the benefits described? |
| Risks/side effects | 19 | Are risks and side effects described? |
| Alternatives | 20 | Are any alternatives described? |

Note. Items were applied to chatbot-generated patient-facing responses. Items that were not applicable to a specific response were excluded from that response-level denominator according to the prespecified EQIP scoring rule.

B2. Modified DISCERN (mDISCERN): 12-item version adopted for AI-generated health information assessment

| **Domain** | **Item** | **Question** |
| --- | --- | --- |
| Section 1: Is the publication reliable? | 1 | Are the aims clear? |
| Section 1: Is the publication reliable? | 2 | Does it achieve its aims? |
| Section 1: Is the publication reliable? | 3 | Is it relevant? |
| Section 1: Is the publication reliable? | 4 | Is it balanced and unbiased? |
| Section 1: Is the publication reliable? | 5 | Does it refer to areas of uncertainty? |
| Section 2: How good is the quality of information on treatment choices? | 6 | Does it describe how each treatment works? |
| Section 2: How good is the quality of information on treatment choices? | 7 | Does it describe the benefits of each treatment? |
| Section 2: How good is the quality of information on treatment choices? | 8 | Does it describe the risks of each treatment? |
| Section 2: How good is the quality of information on treatment choices? | 9 | Does it describe what would happen if no treatment is used? |
| Section 2: How good is the quality of information on treatment choices? | 10 | Does it describe how the treatment choices affect overall quality of life? |
| Section 2: How good is the quality of information on treatment choices? | 11 | Is it clear that there may be more than one possible treatment choice? |
| Section 2: How good is the quality of information on treatment choices? | 12 | Does it provide support for shared decision-making? |

The 12-item mDISCERN version used in this study was adopted from a previously published modified DISCERN instrument for evaluating AI-generated health information. The original DISCERN instrument contains 16 items designed for assessing written consumer health information. Compared with the original version, source-dependent items related to publication references and information attribution were not included because AI-generated chatbot responses do not consistently provide verifiable publication sources. The retained 12 items preserve the core domains of reliability, treatment-related information, balance, and overall information quality. Each item was scored on a 5-point scale ranging from 1 to 5, and the final mDISCERN score was calculated as the mean score of the 12 retained items.

Supplementary Table S9 provides the complete raw chatbot responses for all 432 model-question outputs in a machine-readable CSV format.
