# Supplementary material for: Comparative quality, readability, and factual reliability of four publicly accessible chatbot systems for heart failure patient education: a cross-sectional evaluation
Source: Front Public Health. 2026 Oct 15;14:1966612. doi: 10.3389/fpubh.2026.1966612 (PMC13716745; doi:10.3389/fpubh.2026.1966612)
Supplement: Supplementary file 2 [file Table_1.docx]

**Completed CHART Checklist**

Comparative Quality, Readability, and Factual Reliability of Four Publicly Accessible Chatbot Systems for Heart Failure Patient Education: A Cross-Sectional Evaluation

| **Heading** | **Subheading** | **No** | **CHART checklist item** | **Location in revised submission** | **Completion status** |
| --- | --- | --- | --- | --- | --- |
| Title and abstract | Title | 1a | State that the study is assessing one or more generative AI-driven chatbots for clinical evidence or health advice. | Main manuscript, Title page, p. 1. | Completed |
| Title and abstract | Abstract/summary | 1b | Apply a structured format, if applicable. | Main manuscript, Abstract, pp. 1-2. | Completed |
| Introduction | Background | 2a | State the scientific background, rationale, and healthcare context for evaluating the generative AI-driven chatbot(s), referencing relevant literature when applicable. | Main manuscript, Introduction, pp. 2-3. | Completed |
| Introduction | Background | 2b | State the aims and research questions including the target audience, intervention, comparator(s), and outcome(s). | Main manuscript, Introduction, final paragraph, p. 3. | Completed |
| Methods | Model identifiers | 3a | State the name and version identifier(s) of the generative AI model(s) and chatbot(s) under evaluation, as well as their date of release or last update. | Main manuscript, Section 2.2, pp. 3-4; Supplementary Table S1, Supplementary Material, pp. 2-3. | Completed |
| Methods | Model identifiers | 3b | State whether the generative AI model(s) and chatbot(s) are open-source or closed-source/proprietary. | Supplementary Table S1, Supplementary Material, pp. 2-3. | Completed |
| Methods | Model details | 4a | State whether the generative AI model was a base model or a novel base model, tuned model, or fine-tuned model. | Main manuscript, Section 2.2, pp. 3-4; Supplementary Table S1, Supplementary Material, pp. 2-3. | Completed |
| Methods | Model details | 4b | If a base model is used, cite its development in sufficient detail to identify the model. | Supplementary Table S1, Supplementary Material, pp. 2-3. | Completed where publicly disclosed |
| Methods | Model details | 4c | If a novel base model, tuned model, or fine-tuned model is used, describe the pre- and/or post-implementation/deployment data and parameters. | Not applicable; no novel base model, tuned model, or fine-tuned model was developed. | Not applicable |
| Methods | Prompt engineering | 5a | Describe the evolution of study prompt development. | Main manuscript, Section 2.2, p. 3; Supplementary methods, Supplementary Material, p. 1. | Completed |
| Methods | Prompt engineering | 5ai | Describe the sources of prompts. | Main manuscript, Sections 2.1-2.2, pp. 3-4; Supplementary Appendix A, Supplementary Material, pp. 28-31. | Completed |
| Methods | Prompt engineering | 5aii | State the number and characteristics of the individual(s) involved in prompt engineering. | Main manuscript, Sections 2.1-2.2, pp. 3-4. | Completed |
| Methods | Prompt engineering | 5aiii | Provide details of any patient and public involvement during prompt engineering. | Main manuscript, Limitations, pp. 13-14; no patient or public involvement was used in prompt development. | Not applicable |
| Methods | Prompt engineering | 5b | Provide study prompts. | Main manuscript, Section 2.2, p. 3; Supplementary Appendix A, Supplementary Material, pp. 28-31. | Completed |
| Methods | Query strategy | 6a | State route of access to generative AI model. | Main manuscript, Section 2.2, pp. 3-4; Supplementary Table S1, Supplementary Material, pp. 2-3. | Completed |
| Methods | Query strategy | 6b | State the date(s) and location(s) of queries for the generative AI-driven chatbot(s) including the day, month, and year as well as city and country. | Main manuscript, Section 2.2, p. 3; Supplementary Table S1, Supplementary Material, pp. 2-3. | Completed |
| Methods | Query strategy | 6c | Describe whether prompts were input into separate chat session(s). | Main manuscript, Section 2.2, pp. 3-4; Supplementary Table S1, Supplementary Material, pp. 2-3. | Completed |
| Methods | Query strategy | 6d | Provide all generative AI-driven chatbot output/responses. | Data availability statement, Main manuscript, pp. 15-16; Supplementary Table S9 raw chatbot responses CSV. | Completed with availability statement |
| Methods | Performance evaluation | 7a | Define the ground truth or reference standard used to define successful generative AI-driven chatbot performance. | Main manuscript, Sections 2.3-2.5, pp. 4-6; Supplementary methods, Supplementary Material, p. 1. | Completed |
| Methods | Performance evaluation | 7b | Describe the process undertaken for generative AI-driven chatbot performance evaluation. | Main manuscript, Sections 2.3-2.6, pp. 4-7; Supplementary methods, Supplementary Material, p. 1. | Completed |
| Methods | Performance evaluation | 7bi | State the number and characteristics of team members involved in performance evaluation. | Main manuscript, Sections 2.3-2.4, pp. 4-5. | Completed |
| Methods | Performance evaluation | 7bii | Provide details of any patients and public involvement during the evaluation process. | Main manuscript, Limitations, pp. 13-14; no patient or public involvement was used in performance evaluation. | Not applicable |
| Methods | Performance evaluation | 7biii | State whether evaluators were blinded to the identity of the generative AI-driven chatbot(s) under assessment. | Main manuscript, Section 2.4, p. 5. | Completed |
| Methods | Sample size | 8 | Report how the sample size was determined. | Main manuscript, Sections 2.1-2.2, pp. 3-4; 108 questions x 4 models = 432 responses. | Completed |
| Methods | Data analysis | 9a | Describe statistical analysis methods, including any evaluation of reproducibility of generative AI-driven chatbot responses. | Main manuscript, Section 2.7, pp. 7-8; Supplementary Tables S3-S7, Supplementary Material, pp. 3-27. | Completed |
| Methods | Data analysis | 9ai | Report the measures used for performance evaluation. | Main manuscript, Sections 2.3-2.6, pp. 4-7; Tables 1-3, pp. 19-20; Supplementary Tables S3-S7, Supplementary Material, pp. 3-27. | Completed |
| Results | Results | 10a | Report the performance evaluation undertaken including alignment between chatbot output and ground truth/reference standard using quantitative or mixed methods approaches as applicable. | Main manuscript, Results, pp. 8-10; Tables 1-3, pp. 19-20; Supplementary Tables S3-S7, Supplementary Material, pp. 3-27. | Completed |
| Results | Results | 10b | For responses deviating from the ground truth or reference standard, state the nature of the difference(s). | Main manuscript, Section 3.5, pp. 9-10; Supplementary Table S6, Supplementary Material, pp. 8-24; Supplementary_Table_S6_Hallucination_Audit_Dataset_DHJ-25-1739_R2.csv. | Completed |
| Results | Results | 10c | Indicate where the study protocol can be accessed or state that a protocol was not prepared. | Main manuscript, Data availability statement, pp. 15-16; Supplementary Tables S6 and S9. | Completed |
| Discussion | Interpretation | 11a | Interpret study findings in the context of relevant evidence. | Main manuscript, Discussion, pp. 10-13. | Completed |
| Discussion | Limitations | 11b | Describe the strengths and limitations of the study. | Main manuscript, Limitations, pp. 13-14. | Completed |
| Discussion | Implications | 11c | Describe the potential implications for practice, education, policy, regulation, and research. | Main manuscript, Discussion and Conclusions, pp. 12-14. | Completed |
| Open science | Disclosures | 12a | Report any relevant conflicts of interest for all authors. | Main manuscript, Declaration of conflicting interests, p. 15. | Completed |
| Open science | Funding | 12b | Report sources of funding and their role in the conduct and reporting of the study. | Main manuscript, Funding, p. 15. | Completed |
| Open science | Ethics | 12c | Describe the process undertaken for ethical approval. | Main manuscript, Ethical considerations, p. 15. | Completed |
| Open science | Ethics | 12ci | Describe the measures taken to safeguard data privacy of patient health information, as applicable. | Main manuscript, Ethical considerations, p. 15; no patient data were used. | Completed/not applicable |
| Open science | Ethics | 12cii | State whether permission/licensing was obtained for the use of original, copyrighted data. | Main manuscript, Methods/Data availability, pp. 3 and 15-16; standardized questions were cited from published sources. | Completed |
| Open science | Protocol | 12d | Provide a study protocol. | Main manuscript, Methods, pp. 3-8; no prospectively registered protocol was prepared. | Completed |
| Open science | Main manuscript, Data availability statement, pp. 15-16; S4, S6, and S9 are provided as separate machine-readable CSV files. | 12e | State where study data, code repository, and model parameters can be accessed. | Main manuscript, Data availability statement, pp. 15-16; S4, S6, and S9 are provided as separate machine-readable CSV files. | Completed |
